# Data, Process, and Data-Driven Representations of Digital Twins in Diabetes: Scoping Review

**DOI:** 10.2196/91729

**Published:** 2026-10-07

**Authors:** Beyza Cinar, Louisa van den Boom, Maria Maleshkova

**Affiliations:** 1 Professorship of Data Engineering, Helmut Schmidt University, Holstenhofweg 85, Hamburg, Hamburg, 22043, Germany, 49 17632046601; 2 Department of Neonatology, Clinic for Pediatric and Adolescent Medicine, Helios Klinikum Gifhorn GmbH, Gifhorn, Germany

**Keywords:** data-driven management, data management, digital twin, diabetes, metabolic disease, virtual representation, PRISMA, Preferred Reporting Items for Systematic Reviews and Meta-Analyses

## Abstract

**Background:**

Diabetes is a chronic metabolic condition characterized by impaired blood glucose regulation. It is often linked to serious health complications and comorbidities that significantly affect quality of life, requiring effective management, continuous monitoring, and advanced data analytics. Notably, tailored diabetes management can be enhanced by digital twins (DTs), which serve as adaptive digital representations of patients, using clinical, physiological, and lifestyle data.

**Objective:**

This review explores diabetes-related DTs by examining their patient representation levels. We aim to synthesize the current state of the art and outline the foundations of a holistic, multilevel, multifunctional personalized DT for diabetes management.

**Methods:**

We investigate requirements for a personalized holistic DT and classify existing approaches into three representation levels: (1) data representation, involving structured, context-aware, and AI-ready data architectures that support data analysis, enable semantic interoperability, relationship extraction, and real-time bidirectional data exchange between patient and virtual replica. (2) Process representation, primarily based on mechanistic models simulating glucose-insulin-meal and exercise-glucose dynamics. (3) Data-driven representation, focusing on individualization through predictive modeling of disease onset, adverse events, and the generation of explainable, personalized recommendations. The literature is synthesized to provide a holistic, multilevel perspective on DTs, and to identify research gaps.

**Results:**

DTs accompany patients throughout their lifecycle and span a wide range of use cases, from long-term disease prediction to timely prediction of severe events. However, personalized DTs remain at an early stage of development. Most existing systems primarily function as simulation tools and lack comprehensive integration of data, processes, and data-driven representations. Key gaps include limited use of standardized semantic data models and ontologies, insufficient real-time bidirectional architectures, and fragmented integration of mechanistic and machine learning models, which are often treated as independent rather than complementary components.

**Conclusions:**

Although DTs hold substantial potential to advance personalized diabetes care, current implementations remain fragmented and incomplete. Future research should prioritize the development of holistic, multilevel DTs that integrate interoperable data infrastructures, mechanistic simulations, and data-driven models into cohesive, personalized systems capable of supporting lifelong disease management.

## Introduction

### Diabetes and Related Complications

Diabetes is one of the most prevalent chronic diseases worldwide and is projected to affect 853 million people by 2050, with incidence rates also rising among children [1,2]. The disease etiology classifies diabetes into different types. Type 1 diabetes (T1D) is an incurable autoimmune disorder in which insulin-producing beta-cells are destroyed, resulting in absolute insulin deficiency and the need for lifelong exogenous insulin therapy [3]. T1D is more frequently diagnosed during childhood, when age-related physiological differences can introduce additional clinical challenges [4,5]. Type 2 diabetes (T2D) is typically characterized by insulin resistance and relative insulin deficiency [6]. The risk of developing T2D increases with age, obesity, and lack of physical activity (PA) and is further associated with genetic predisposition, epigenetic changes, inflammation, metabolic stress, and chronically elevated blood glucose [3,6-8]. Over time, the condition can progress to an insulin secretory defect with insulin resistance requiring external insulin treatment [3,6]. Prediabetes is characterized by elevated glucose levels, impaired fasting glucose, or impaired glucose tolerance below the diabetic threshold and has an increased risk of diabetes onset [6]. Given the differences in pathophysiology across diabetes types, individualized treatment is recommended. Tailoring therapy to each patient is also essential, as standardized clinical guidelines, glycemic thresholds, and recommended target ranges may not capture interindividual variability in glucose dynamics [3,9,10].

Glycemic status is typically categorized into distinct ranges. Diabetic glucose is defined as hyperglycemia (>180 mg/dL), while the target range is 70‐180 mg/dL [11]. A common severe complication in T1D and insulin-treated T2D with impaired insulin production is hypoglycemia. Hypoglycemia (<70 mg/dL) and severe hypoglycemia (<54 mg/dL) often occur due to rapid declines activated by insulin therapy [11,12]. Severe hypoglycemia is clinically significant and is further defined by the need for third-party assistance [12]. Hypoglycemia can cause distress, dizziness, and loss of consciousness. The symptoms can be autonomic and neuroglycopenic. As hypoglycemia is often asymptomatic and may occur during sleep, it is associated with increased mortality [12].

Diabetes is commonly accompanied by comorbidities, influenced by age, diabetes duration, insulin usage, and glycemic variability [3,13,14]. Comorbidities require additional care and restrict medication options, as certain conditions increase hypoglycemia risk. This underscores the importance of context-aware management [7,10,15,16]. In particular, hyperglycemia is associated with vascular complications in T1D and T2D [9,17,18]. Up to 75% of adults with T2D are reported to have hypertension, which significantly elevates the risk of cardiovascular disease (CVD), nephropathy, retinopathy, and mortality [19-21]. In addition, a family history (FH) of autoimmunity in patients with T1D is associated with an increased risk of thyroid disease, celiac disease, and gastritis [18,22]. Hence, to prevent comorbidities and maintain target glucose levels, frequent monitoring, a healthy lifestyle, and weight control are crucial [5,23]. Management is especially challenging for children, youth, and older adults. Children depend on caregivers [24,25], and their therapeutic response differs from that of adults with diabetes, whereas older adults face geriatric conditions and comorbidities [10,23]. Common complications are summarized in Figure 1.

**Figure 1.**
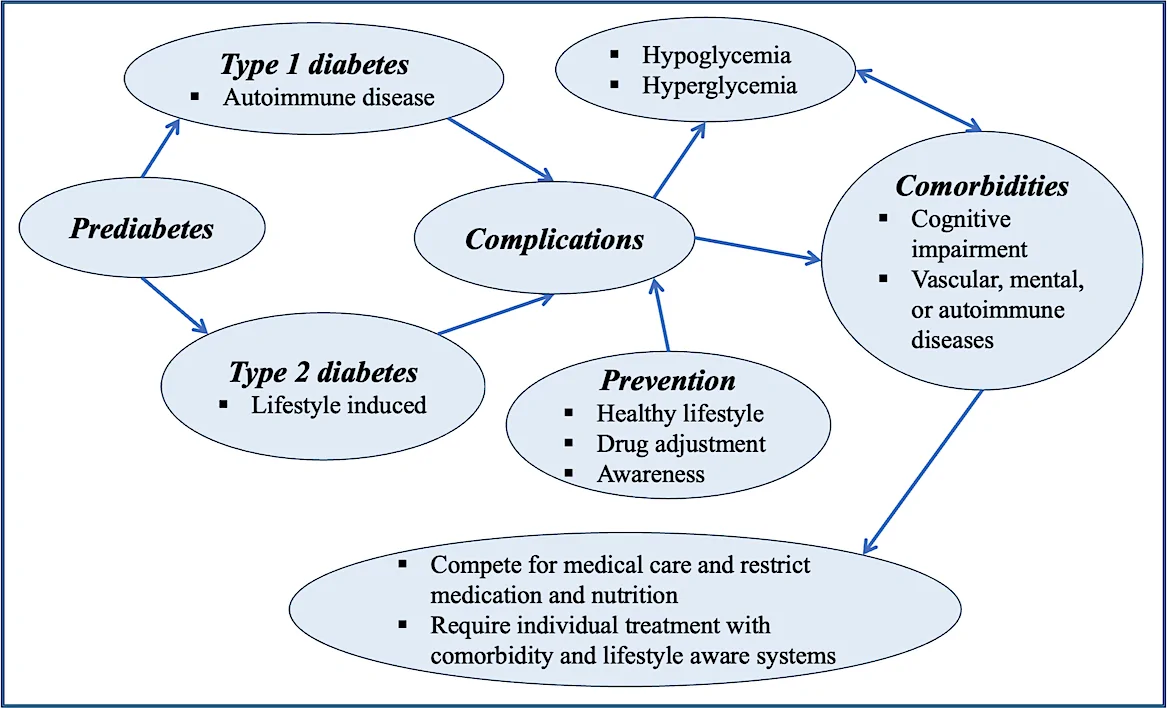
Diabetes and its complications.

### Digital Twins for Personalized Diabetes Management

Continuous glucose monitoring (CGM) devices improve glucose control and self-awareness [3]. Their utility can be further enhanced through data analytics, the identification of patient-specific patterns, and the prediction of adverse events [26]. Clinical studies suggest that individual, technology-supported guidance delivered by health care professionals (HPs) is associated with improved glycemic control, weight reduction, and reduced side effects [27-30]. However, conventional approaches based on mathematical models, isolated simulations, or sensor-driven predictions often fail to capture patient heterogeneity and the clinical context, neglecting medical knowledge, patient history, and individual preferences.

Digital twins (DTs) address these limitations by accompanying patients throughout their life cycle, dynamically adapting to real-time patient data, incorporating new diagnoses, and tailoring care to individual needs. DTs are virtual replicas of physical entities that leverage digital approaches and real-time data exchange to mirror and advance their physical counterparts [31-33]. They are self-aware, intelligent counterparts that form a central knowledge base aggregating various abstraction levels [34]. Accurate virtual representations are created by integrating historical, contextual, static, and real-time data [35]. In the medical domain, the concept of human digital twins (HDTs) was introduced in 1993, which proposed an ontology that progresses from high-level organs to tissues, cells, and proteins [36,37]. HDTs adapt to an individual’s context, disease trajectories, environment, and behavior [38]. Use cases include virtual trials, simulations, disease detection, progression tracking, and personal treatment optimization [25,39-41].

As DTs emerge as a promising technology in health care, supporting telemonitoring and self-management [35,42,43], this study investigates current advancements in their application to diabetes and proposes a perspective on multilevel DT systems for diabetes. Previous reviews have primarily focused on key techniques and the potential of DTs in diabetes [44], the structure, operating conditions, and characteristics of DTs for T1D [43], and key characteristics and modeling strategies for metabolic diseases [42]. These reviews identified a research gap in multifunctional multilayered DTs. However, they do not provide a comprehensive analysis of their functional capabilities and modular components. To advance the field, this review systematically evaluates current developments in diabetes-related DTs with a specific emphasis on personalized representations. Based on existing definitions and requirements, we propose a DT framework composed of 2 modules (data and model) and 3 representation levels [35,38,42,45,46]. The data module integrates data and mirrors the physical patient. It manages storage, preprocessing, and knowledge representation, and uses unsupervised and semantic methods [35,45-47]. In addition, continuous data flow with a bijective relationship enables communication between both entities, ensuring real-world adaptability [35,44]. The model module comprises 2 submodules: the process and data-driven representations [45]. The process representation consists of mechanistic models, usually based on ordinary differential equations (ODEs), that simulate biological processes and provide explainability [23,42,44]. Data-driven models leverage data analytics and AI to predict adverse events and disease risk. They also optimize and personalize performance [23,42,48]. A holistic DT sustains its lifecycle through a dynamic structure and feedback layer, ensuring continuous refinement, adaptation to the patient’s context, and optimal model performance [35,38].

### Aim of This Study

This literature review provides a comprehensive overview of diabetes-related DT systems by synthesizing state-of-the-art DT approaches, DT-enabling components, and intermediate architectures relevant to T1D, T2D, nutrition, and metabolism. Specifically, we investigate the components and functionalities required to enable a holistic, personalized, and adaptive DT for diabetes. To structure the review, studies are classified into data-based, process-based, and data-driven representations. Thus, their patient representation level and degree of personalization are assessed as shown in Figure 2. Patient representation refers to the personalized virtual representation and the structured organization of data representing or extracted from humans.

**Figure 2.**
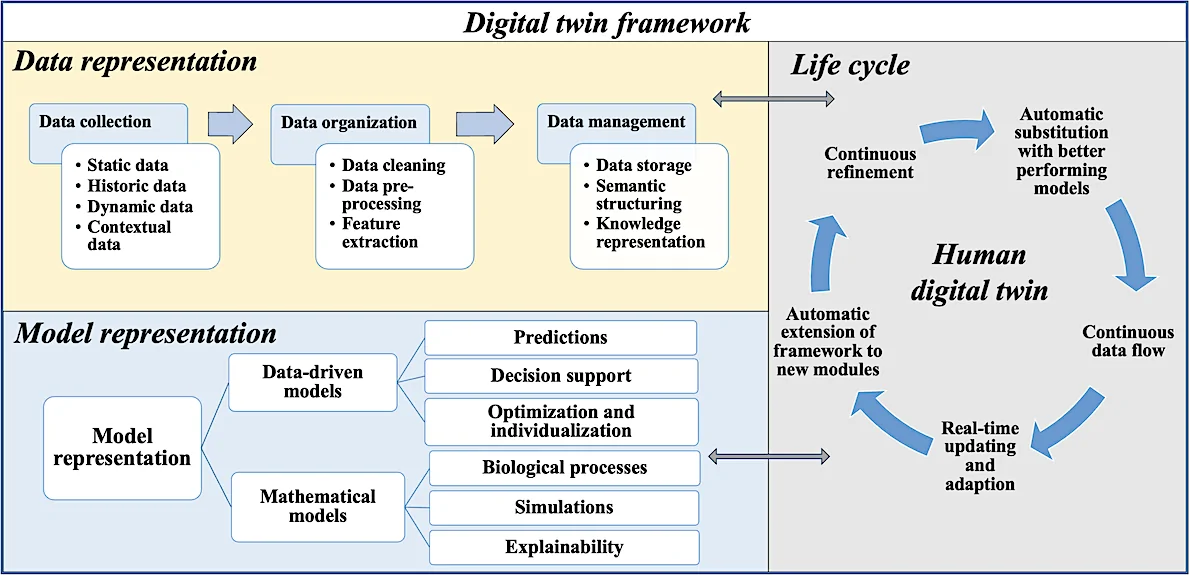
Illustrative framework describing the concept of a closed-loop digital twin.

Building on this categorization, we analyze the literature based on the following questions: (1) What are the main characteristics of diabetes-related DTs? (2) What are potential input features, and how is data acquired, stored, or structured for the data representation layer? (3) What are the use cases and submodules of the model layer, and how are these differentiated for process and data-driven representations? and (4) How are patients represented?

The remainder of this work is organized as follows: the Methods section outlines the methodology, and the Results section presents the results, categorized into data, process, and data-driven representations. Finally, the Discussion section discusses the findings and highlights research gaps, while the Conclusions section provides a conclusion to this review.

## Methods

### Study Design

This scoping literature review presents the current state of the art of DTs in diabetes. The review aims to propose a framework for holistic DTs by integrating insights from current research. We collect studies describing multilevel and fully integrated DT systems to depict the architectures and use cases of holistic DTs in diabetes. In addition, we extract information on intermediate DTs and DT-enabling systems to identify the components required for holistic DTs, encompassing 3 integration levels of data, process, and data-driven representations.

Titles, abstracts, and full texts were manually screened by the primary reviewer according to predefined inclusion criteria and study objectives. Additionally, eligible studies were manually charted according to predefined representation-domain criteria. No protocol for this scoping review was registered.

### Information Sources and Search Strategy

This review is reported according to the PRISMA-ScR (Preferred Reporting Items for Systematic Reviews and Meta-Analyses extension for Scoping Reviews) method [49,50]. Studies were searched in “Scopus,” “PubMed,” “IEEE Xplore,” and “Google Scholar” with the following search term: ((“virtual representation”) OR (“digital twin”)) AND (“diabetes” OR “metabolic” OR “nutrition” OR “exercise”). The search strategy was developed through iterative pilot testing of multiple keyword combinations and synonyms related to DTs (eg, insulin or virtual model). As broader search terms yielded many nonrelevant studies, the final search string was selected to optimize the balance between sensitivity and relevance. Terms such as “physiological model,” “in silico,” “artificial pancreas,” “glucose-insulin model,” “personalized model,” and “metabolic simulator” were not explicitly searched for, as they encompass a broader body of simulation and modeling literature beyond the intended DT-focused scope of the review. Studies using such approaches were included when they emerged within the retrieved literature or were identified through reference tracking, because they represent subcomponents of fully integrative (holistic) DT systems. To maintain methodological consistency, the same search strings were used across all databases. Hence, the search query had to comply with the keyword and query-length restrictions of Scopus.

The search was conducted in August 2024, and search alerts were activated on September 1, 2024. The search in “IEEE Xplore” and “PubMed” was not filtered, while it was limited to document types of only papers, conference papers, and book chapters in “Scopus.” Additionally, the language was restricted to only English and German. The search was further filtered to 2010 onward, as “PubMed” and “IEEE Xplore” did not return any studies before then. In particular, “Scopus” and “PubMed” showed a peak in 2023, with most papers published in 2024 highlighting the research field’s hot topic. The search strategy is summarized in Multimedia Appendix 1.

### Eligibility Criteria

The primary focus of the search was on enhancing personalized treatment and diabetes self-management through DTs. Studies were included if they addressed data, process, or data-driven representations within the DT framework, but inclusion did not imply classification as a holistic DT. Given the aim of providing an overview of potential DT implementations, preliminary works and conceptual studies were also considered. While simulators were not explicitly searched for, relevant studies foundational to DT development were included, primarily through reference tracking. The review specifically targeted chronic diabetes management and prevention, with a focus on T1D, T2D, and prediabetes, while prioritizing personalized DT approaches. Studies addressing clinical applications for physicians were included if they represented relevant submodules of DTs integrating clinical diagnosis, comorbidity prediction, and prevention, also providing insights for patients. In addition, studies on metabolic twins were considered because their findings can be translated to patients with diabetes and serve as an important foundation for holistic DTs.

In contrast, studies exclusively addressing gestational or neonatal diabetes, or involving intensive care unit patients without specifying the type of diabetes, were excluded. Research focusing on malnutrition without a direct association with diabetes was also removed. DT models developed exclusively for drug testing, pharmaceutical evaluation, or clinical trial simulation were not included during screening. In addition, studies addressing conditions such as stroke, thyroid disease, dermatological diseases, mental health conditions such as schizophrenia, and Parkinson disease without a clear relevance to diabetes within the title or abstract were excluded. Likewise, fitness and sport-related studies were only included if a clear relation to diabetes was described. Furthermore, studies focused on the visual representation of anatomical structures, such as the pancreas, or on retinal imaging were excluded, as they do not directly contribute to personalized diabetes management and often require additional medical systems. The literature reporting extended reality or image-based representations was removed because these primarily serve educational purposes or physician-oriented treatment planning.

### Conceptual Scope and DT Categorization

The included studies were categorized according to their representation levels within 3 predefined categories. A study was classified as data representation if it included acquisition, storage, management, or knowledge representation [45,51,52] of patient-specific and domain data. A process representation was assigned when mathematical, biochemical, or mechanistic models were used to simulate biological processes relevant to diabetes. A data-driven representation required AI or data analytics applied to patient-specific data for personalization, optimization, prediction, and decision support.

Studies implementing a single representation level were categorized as DT-enabling components. For our classification framework, a DT at least needs a virtual representation of the data and relationships, as well as a process representation of the system’s mechanics or biochemistry. Finally, for human-related DTs, a personalization layer, best achieved through data-driven representation, is required. Consequently, a fully integrated system requires the intersection of data representation, model representation, and personalization.

### Data Charting

For each included study, the following information was manually extracted: (1) publication information; (2) research focus: diabetes type; (3) information on data, process, and data-driven representations; (4) methods used for representation; (5) representation level: single, dual, and triple; (6) inputs used for representation; (7) optimization methods for process representation; (8) AI- and machine learning (ML)–based data-driven prediction models; (9) personalization mechanisms; (10) clinical application; and (11) method validation.

Extracted information was synthesized according to the predefined representation framework. Dual- and triple-based DT systems were analyzed in more detail to outline the current state of the art.

### Quality Assessment

We did not perform formal risk-of-bias or evidence-quality assessment because the primary objective of this review was conceptual mapping and integrative representation synthesis rather than quantitative evaluation of intervention efficacy or comparative clinical performance.

## Results

### Overview

This review explores the state of the art of DTs for diabetes, including relevant work on nutrition management. Selected studies are classified based on the abstraction levels of data-based, process-based, and data-driven representations. Finally, promising results are highlighted while also investigating continuous refinement, the research state, and use cases of the applications.

### Overview of the Included Literature

The PRISMA (Preferred Reporting Items for Systematic Reviews and Meta-Analyses) flowchart is illustrated in Figure 3. A total of 51 studies were included in this review. Analyzing this study’s cohort, T1D and T2D are covered almost equally, with 39% (20/51) and 43% (22/51), respectively, with some studies including both diabetes types. Additionally, 20% (10/51) of studies do not specify the type of diabetes or generically focus on chronic diseases. Lastly, diabetes prevention by targeting prediabetes and obesity is studied by 10% (5/51) of selected approaches. Regarding the primary applications of DTs, intermediate DTs, or DT-enabling components in diabetes research, which are summarized in Table S1 in the Multimedia Appendix 2 [5,7-9,14,23-25,37-40,47,48,53-74], 54% (15/28) of studies simulate glucose levels, while 36% (10/28) focus on enhancing precision insulin therapy through data-driven adjustments or simulation-based optimization algorithms. Lifestyle recommendations, including decision support for nutrition or exercise, account for 43% (12/28) of studies, indicating a balanced distribution. Lastly, 25% (7/28) develop diagnostic tools for assessing disease onset and progression, and 29% (8/28) of studies incorporate education. As shown in Figure 4, relatively few studies were published until 2022, with a notable rise in publications in 2023 and 2024, underscoring the growing relevance of this research field.

The reviewed literature demonstrated differences in the operationalization of DT-related concepts. Most identified studies implemented isolated mechanistic, predictive, or representational components rather than fully integrated holistic DT architectures. Usually, conceptual frameworks and simulation-based approaches are presented.

**Figure 3.**
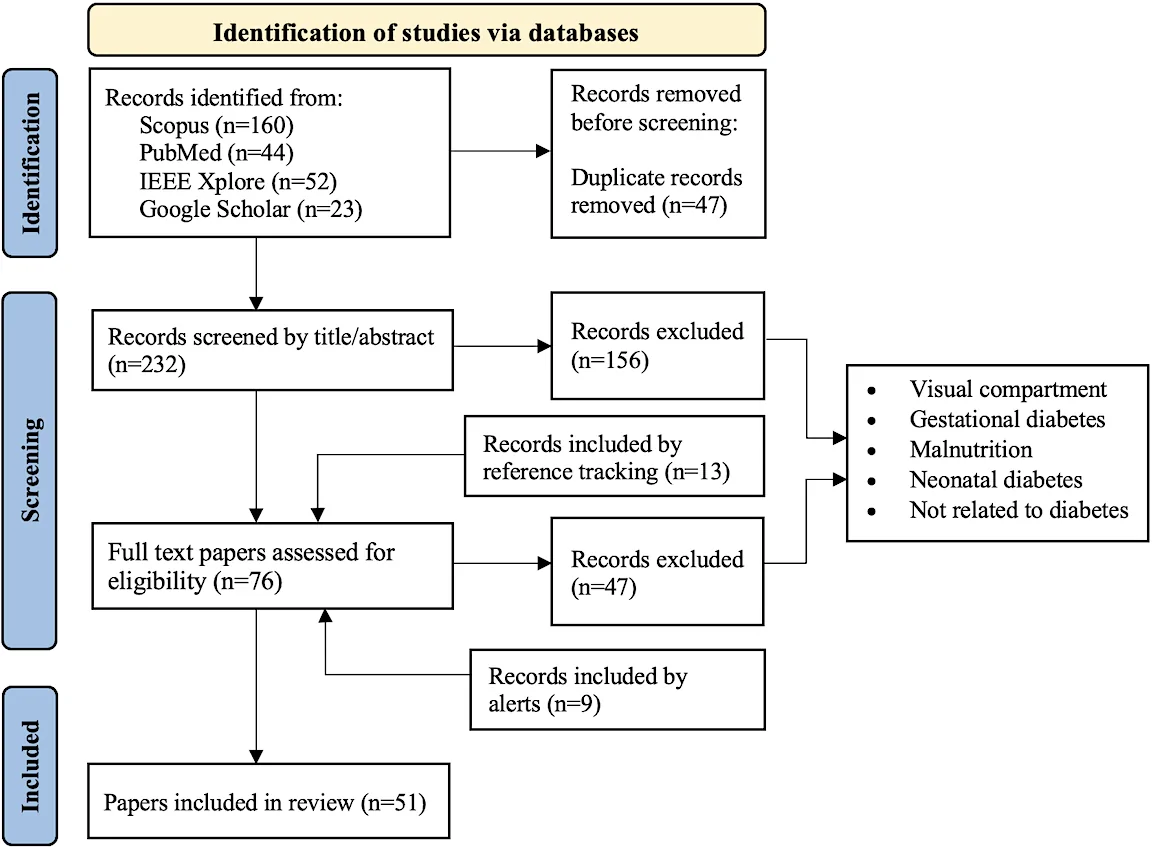
PRISMA (Preferred Reporting Items for Systematic Reviews and Meta-Analyses) flow diagram of the search methodology.

**Figure 4.**
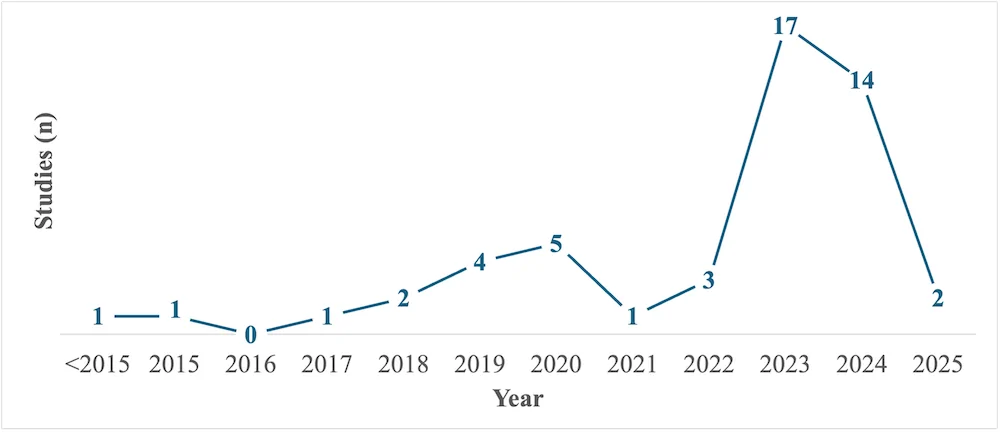
Numbers of studies published per year.

### Data Representation

#### Overview

Data are defined as the brain of the DT and are essential for virtualization [45]. Cross-domain literature reveals that the data layer encompasses data collection, organization, and management, and represents domain knowledge [45,51,52]. Furthermore, data compatibility with AI-driven predictions and decision-making processes should be ensured [23]. AI-readiness involves transparent data collection, preparation, quality assurance, and documentation, providing data as machine-readable metadata [75,76].

This section explores existing standards, collected data, organizational strategies, and storage solutions specifically tailored to diabetes research. Through an integrative synthesis of DT-related approaches, we illustrate how the data layer of a holistic DT for diabetes could be structured.

#### Data Collection

Combining the methods and use cases identified in the literature, a holistic DT for diabetes should integrate data from diverse sources, including routinely available clinical test results, electronic health records (EHRs), and wearable sensor data (eg, glucometers, CGM devices, smart scales, sphygmomanometers, activity trackers, calorie calculators, and insulin pumps) [9,14,25,27,47,48,53]. In particular, historical and real-time context (eg, location, activity, time, stress, and vital parameters) should be aligned. For a comprehensive patient profile, the literature emphasizes collecting data on diabetes history, FH, comorbidities, behavior, and medication [23], especially taken up to 3 years before diagnosis [8,30]. For diet management, studies commonly collect the type, amount, and frequency of consumed food, as well as metabolic, activity, and emotional data. To increase personalization, the importance of integrating these variables with genetic information is highlighted [53,54], alongside incorporating patient taste preferences, allergies, and individual goals [55,56]. Additionally, personalized, adaptive diet planning that considers comorbidities is enabled by representing knowledge of different diets, macronutrient combinations, and fasting schedules [54]. Finally, Vaskovsky et al [55] propose mapping food components to their own DTs, increasing semantic context.

Regarding the use of Internet of Things (IoT) and mobile devices, several studies propose mobile health care or virtual data platforms linked to smart devices and wearables, enabling automatic, continuous data acquisition and integrated analysis [9,27,39]. Lee et al [9] present a government-supported big data exchange platform in which data are stored on personal cloud services or smartphones and can be collected from health care apps, log data, and IoT devices. In addition, a platform to upload clinical and genetic data is provided. The platforms can also integrate different data modalities, such as images of meals or retinal data [39,57]. Retinal images can be collected using IoT devices and smartphones [57]. Moreover, Sai et al [39] enhance data exchange platforms with nonfungible token technology to incentivize data sharing through monetary rewards. To ensure privacy, patient data are anonymized using generative adversarial networks (GANs). These platforms enhance self-control over data, improve glucose management, and increase awareness about lifestyle impact. Moreover, clinicians can monitor patients within a comprehensive contextual framework [9,27,39].

Regarding input data, the distribution of input data across all studies is shown in Table S2 in Multimedia Appendix 2 summarizes commonly used data from the included studies. Glucose and carbohydrate values are the most frequently monitored features, with 69% (20/29) and 72% (21/29), respectively. Glucose data are categorized into CGM data, which are more often incorporated into data-driven frameworks, and self-glucose blood measurements obtained from blood tests and finger pricks, which are more common in mechanistic models. Only half of the studies using carbohydrate data collected protein (10/29, 34%) and fat components of the meal (9/29, 31%). Insulin and PA are regularly tracked, with 52% (15/29) and 38% (11/29), respectively. Often, demographics (12/29, 41%), EHRs (9/29, 31%), and vital signs (9/29, 31%) are considered for personalization, besides wearable data. However, drug use, images, genetic data, and laboratory test results are seldom explored, with less than 6 studies each, limiting the potential for personalized care and for models to learn contextual information.

#### Data Organization

Regarding data cleaning, one submodule of the DT needs to focus on data preprocessing, including data cleaning, outlier detection and correction, imputation of missing values, and extraction of reliable information [58,77]. For imputation, studies commonly use forward filling, averaging, mathematical approaches, or data-driven methods such as linear interpolation, Multiple Imputation by Chained Equations, or logistic regression [23,56,59]. For metabolic values, missforest imputation is proposed [25].

Literature also highlights the importance of addressing uncertainty and variance. Proposed methods include pooling data using Rubin rules [56,78] or correcting CGM data errors with calibration-dependent parameters and autoregressive noise modeling [79]. Data reliability can be further ensured through validation and error correction via user feedback and automated detection of historical trend deviations [56,78]. To mitigate the problem of small and imbalanced datasets, synthetic data generated with GANs are fused with real data [80].

Regarding feature extraction, after data cleaning, features can be extracted, and behavior can be automatically retrieved from measured IoT data. These features can improve the performance of subsequent models of the other representation levels. For instance, studies specify the activity type (eg, standing, sitting, walking, or unknown) [23,60] or categorize lifestyle into busy, reasonably active, lightly active, or lethargic based on a weighted threshold comparison, if more context is available (eg, calorie, consumption, step count, distance, sleep, weight, and active minutes) [39,60]. From time-series data, recent trends, long-term patterns, and cohort-based models can be extracted [56]. Thamotharan et al [23] use structural time-series analysis (TSA) to decompose time-series data. They also estimate the possible prediction performance of various features with an autocorrelation or partial autocorrelation function, showing that time-varying intermittent behaviors, causal variables, and exogenous factors influence predictions of blood glucose levels (BGLs) [23]. Additionally, they create matrix profiles, storing the distance between each subsequence within a time series and its neighbor, to discover intrapersonal and population-based patterns and anomalies that impact the trajectory of BGLs [23]. Text data from EHR can be processed with pretrained natural language models such as ClinicalBERT [8,61].

In particular, diet information and nutrition knowledge can be organized differently. Patients can be grouped by diet scheme (eg, ketogenic or low-fat and low-calorie) to generate different recommendations [62], or dietary habits, nutritional content, and patient profiles can be extracted from EHRs and lifestyle data [63]. A commercial example is Twin Health, a DT platform for T2D that enables patients to manage their diet and glucose levels. The platform integrates a food database, from which the user selects consumed food components, and the user must input the type and quantity to extract nutritional data (eg, calories, macronutrients, glycemic index, and glycemic load). Impactful extracted features are stressed to be carbohydrate-to-protein ratio, time since the last meal, and glucose trends [28,30,56,81]. Other approaches include automatic detection of meal and meal-related context, such as meal timing from raw glucose and insulin data [77] and estimation of carbohydrate and nutrient intake from food images (eg, using the VGG-16 (Visual Geometry Group 16 Layer) or a convolutional neural network) and meal quantity or recipe data [23,27,60,64]. These models can be refined by user feedback, behavioral inferences, alerts, and context to enhance personalized recommendations [23,56]. To streamline data collection, the literature proposes using heuristic-based event labeling to automatically categorize events and generate context [5]. Finally, food can be digitally replicated using mathematical models and embedded food sensors that extract nutritional data. These replications can interact with the HDT, enabling precise dietary recommendations while structuring food to align with health needs [63].

Regarding data fusion, after collecting all available data, the data are fused and integrated. Wang et al [61] further divide daily data into 7 time periods based on pre- and postmealtime and bedtime. Then, for each time period, feature vectors are created with the fused datasets. Transformers are further approaches to encoding patients as vectors.

#### Data Management

To ensure holistic representations, contextual, historical, and stream data must be efficiently processed using robust infrastructures [35]. The data storage framework and architecture primarily rely on Internet of Medical Things (IoMT) technology, edge computing, and cloud computing. Edge computing reduces latency and enables real-time decisions. Cloud computing optimizes resources to achieve variable-cost-driven access and ensure effective service delivery [80]. A typical architecture using IoMT technology can comprise a communication, perception, transfer, and application module. The perception layer includes the sensors, while the transfer layer facilitates data exchange via edge nodes, storing information locally in an SQL database and syncing with a NoSQL cloud database. A web interface visualizes patterns, and the application layer implements the DT on the edge. Additionally, conceptual approaches emphasize 3 core requirements to aggregate real-time data. First, automatic detection and integration of available physical devices within a network is required, typically facilitated with mobile apps. The second is interoperability, enabling seamless detection, composition, and synchronization of DTs across domains for efficient data exchange. This can be enabled by using a cloud-based, multipurpose backend service that stores data in a permission-based SQL database. The third is fidelity, ensuring accurate representation of the physical entity through dynamic data collection, processing, and contextual updates. A context-monitoring feedback loop refines health status using adaptive structural models [5,34].

Regarding data modeling, data modeling defines the data concept, requiring semantic structuring and a shared vocabulary to represent domain knowledge [78]. For instance, ontologies represent entities in machine-readable form and can model domains using knowledge graphs (KGs) [45]. For DTs in diabetes, studies use ontologies and KGs to provide templates for mechanistic models, identify causal relationships, and extract disease-related features for ML applications. They can identify relationships between biomedical entities (eg, genes, proteins, metabolites, drugs, and clinical phenotypes) and diagnose health states. In addition, disease progression can be modeled by exploring indirect links among predictive features and diseases [25,47]. Across the literature, personal KGs are generated from EHR and continuous wearable data. Proposed frameworks support dynamic and bidirectional data mapping using the global-local-as-view architecture. Mapping accuracy can be further improved by refining data integration with conditional random fields [47].

Besides patient profile KGs, generalized metabolic flux (GMF) maps can be created, in which metabolites are grouped into generalized fluxes based on biological processes. Nodes represent key components such as glucose and lipid metabolism, metabolites, and physiological parameters, while edges represent the generalized fluxes. The framework can model metabolic conditions as outcomes of lifelong changes in metabolic fluxes driven by biomolecular pathways and gene networks. Surian et al [40] and Batagov et al [48] also use statistical methods, including the Wilcoxon-Mann-Whitney test, to analyze associations among metabolites, flux vectors, and diabetic complications. Then, path queries, profile comparisons, and correlation analysis identify features linked to specific complications and disease progression, such as retinopathy, ophthalmic complications, and chronic kidney disease (CKD). Time-to-event analysis further reveals differences in progression rates across risk categories (eg, low, moderate, and high severity).

KGs can also support explainability by revealing relationships between predictive factors and disease outcomes. For instance, the relationship between weighted features and disease progression can be explained using graph analysis. Zhang et al [25] derive a subgraph from the Scalable Precision Medicine Open Knowledge Engine to connect the most predictive features and the T2D-related node. Scalable Precision Medicine Open Knowledge Engine is an ontology integrating data from various biomedical databases across diverse domains [82]. Additionally, the Topic PageRank algorithm highlights graph features related to the target features of interest [25]. The network parsimony-based shortest-path method can further reveal insights into associated drugs and proteins in patients with T2D [25].

Finally, the framework should adopt unified medical standards, such as HL7 (Health Level Seven), the international standard for clinical software that enables the exchange and communication between medical devices or applications.

### Process Representation

#### Overview

Process representation models physiological and biological processes using mathematical and statistical methods. Studies usually rely on mechanistic models that use mathematical functions and data assimilation to simulate metabolism, allowing parameter adjustments for therapy titration and the identification of changes in biological features [24,25,42]. Their key advantage is interpretability, as the relationship between input parameters and outputs can be displayed, improving awareness [42,54,83]. While most proposed models are hybrid and iteratively refined using data-driven optimization functions, we do not consider them data-driven because they primarily rely on mathematical functions.

This section synthesizes the process representations identified across the literature and illustrates how biological processes are modeled for diabetes-related DT systems. First, individual simulation modules are presented. The simulation and optimization methods are then depicted in detail. The synthesis is based on all the retrieved literature and does not separate between diabetes types. However, practical implementation of the process layer should be tailored to individual patient characteristics, disease state, and diabetes subtype.

#### Modules

The literature reveals that diabetes-related DT simulators model key processes of insulin, meal, and exercise impacts on glucose through various submodules, as shown in Table S3 in Multimedia Appendix 2. The modules can be classified into insulin, glucose, and glycogen, drug, exercise, the inflammatory system, the gastrointestinal tract, and metabolism. The following synthesis integrates the identified modules from the literature to illustrate the possible components of a holistic metabolism for a diabetes-related DT. The individual studies usually model only a subset.

Regarding the insulin module, it models insulin kinetics, dynamics, resistance, and sensitivity across tissues to simulate insulin requirements and their impact on glucose levels. Insulin kinetics determine plasma concentration, while dynamics describe its glucose-regulating effects, based on insulin mass, infusion rate, absorption time, sensitivity, and effectiveness [84]. This module can model basal and bolus insulin inflows separately, typically using nonlinear differential equations [85]. Across the reviewed literature, insulin processes are represented at multiple biological abstraction levels, ranging from physiological compartment models to cellular levels. Insulin inflow into the plasma is modeled from pancreatic secretion, while outflow is represented by liver clearance [54,85]. In particular, short-acting insulin is absorbed into the plasma, and time lags are estimated using coupled delay equations [85]. In the interstitial fluid, insulin inflow matches plasma outflow, while insulin outflow reflects cellular uptake [85]. Insulin resistance and diabetes progression are primarily modeled using ODEs at the tissue and cellular levels. These models incorporate organ-specific glucose uptake, intracellular signaling, protein expression, and adiposity-related changes [7]. A subcutaneous tissue compartment comprises absorption, dissociation, clearance, and distribution volume [79] and accounts for the time delay between insulin administration and its appearance in the blood [82]. Additionally, several studies model daily variations in insulin sensitivity using time-varying parameters, a variability control signal to capture circadian changes, or insulin-dependent glucose use and insulin action on endogenous glucose production [37,85,86]. To account for differences in insulin dynamics across diabetes types and disease stages, β-cell insulin production is set to 0 in models for T1D, reducing the dynamics to insulin absorption alone [65,85].

Regarding glucose and glucagon modules, this module simulates glucose dynamics integrating mechanisms of meal absorption rate, insulin-glucagon actions, and nonlinear glucose responses to hypoglycemia, including counterregulation [84,86]. Likewise, glucose inflows and outflows are modeled through different representations, including usage in fat, muscle, and adipose tissue. Moreover, in the gastrointestinal tract, studies simulate glucose appearance, renal and liver production, storage, and uptake [7,54,86]. In particular, nonlinear coupled differential equations can describe compartments such as the gut, plasma, interstitial fluid, and subcutaneous tissue [85]. Plasma glucose balance includes gut inflow, liver production, tissue uptake, and renal clearance, with insulin-dependent and independent uptake following Michaelis-Menten kinetics. Exogenous insulin inflow from short- and long-acting injections is also included by various studies [85,87]. The literature models hepatic glucose production using hyperbolic functions with asymptotic maximum rate or ODEs [65,87]. Context to improve CGM-based simulations can be further provided by an intestinal model that accounts for glucose diffusion from plasma into the interstitial space [66,79,86]. The interstitial glucose kinetics are described using a single-compartment linear model, adjusted for plasma-interstitial glucose gradients [79]. Glucose kinetics output the glucose absorption rate from meals, incorporating actions of glucose absorption, glucose-insulin kinetics, and glycogen kinetics and dynamics [77]. The glucagon submodule represents glucagon absorption, plasma action, clearance, secretion, and sensitivity [84,86].

Regarding the drug module, diabetes can be accompanied by multiple comorbidities, each with its own medication plan. For instance, weight-reducing drugs such as topiramate can be included in simulations of weight-related meal dynamics. One study proposes modeling topiramate response at tissue levels using ODEs within a compartmental pharmacokinetics model, in which energy intake is altered in response to the drug dose [7]. Other drug interactions remain underexplored.

Regarding the inflammatory system module, it can simulate insulin deficiency by modeling gene-regulatory mechanisms that lead to the destruction of insulin-producing beta cells [88]. For instance, it is proposed to simulate dynamic hormonal and immune responses using a multiscale discrete immune system model, in which key immune cells are represented as distinct tissues within a cellular immunology framework, each modeled as an agent. These are governed by rules describing responses to pathogens [88,89]. The module also examines the interplay between adipose tissue, inflammation, and diabetes, particularly in the context of high-calorie meal intake. It describes adipose tissue growth, leading to fat accumulation, the production of proinflammatory cytokines, and the development of an inflammatory state [88].

Regarding the activity module, it represents the personalized effects of PA on hormone regulation, interleukin-6 secretion, and weight changes [8]. Exercise is usually quantified by volume of oxygen, with the model simulating oxygen consumption, epinephrine, and glucagon-insulin secretion dynamics. Additionally, hormone and glucose reactions to different exercise types (eg, cycling, walking, running, and stepping) can be simulated [90]. In particular, the literature suggests that exercise types, such as resistance and cardiovascular training, should be modeled separately [24]. Plasma interleukin-6 dynamics, influenced by variations in oxygen uptake during exercise and by skeletal muscle and adipose tissue secretion, can be described using ODEs, with oxygen consumption serving as a measure of exercise intensity [91,92]. Studies also describe epinephrine release as it is correlated with pancreatic insulin and glucagon secretion [90]. Consequently, the exercise model can model changes in insulin sensitivity impacted by increases in peripheral insulin, liver and glucose production, active tissue glucose uptake, oxygen consumption, and active muscle mass [84].

Regarding the gastrointestinal tract module, this module models the effects of gastric emptying and meal composition on glucose metabolism and weight dynamics, which can be described using ODEs [7]. Stomach emptying is differentiated between glucose leaving the stomach, glucose in the jejunum, and glucose in the ileum [65,87]. For mixed meals, studies simulate the appearance rates of glucose, alanine, and triglycerides based on carbohydrate, fat, and protein intake [92]. Meal responses can be modeled as metabolic flows between organs, accounting for meal frequency, composition, ingestion rate, and body weight [54]. In particular, postprandial glucose dynamics incorporate delays between digestion and plasma glucose appearance [67,85]. The module also incorporates gut absorption, glucose-insulin and glucagon-insulin hormonal dynamics, glucose usage in different organs and tissues, hepatic glycogen storage, protein metabolism, and long-term metabolic dynamics [54,92]. The gastric emptying model delivers glucose to the gut, and the gut compartment accounts for nutrient absorption efficiency, appearance rates, and effective distribution volume [85,92].

Regarding the metabolism module, it simulates the glucose-insulin dynamics, integrating hepatic and renal responses. Across studies, key parameters include insulin action, glucose appearance rates, insulin sensitivity, and glucose-insulin meal interactions [37,54,79,87]. The liver compartment describing hepatic glucose metabolism includes a storage module that provides a continuous glucose supply, using glucose appearance rates from the gastrointestinal module [67]. Furthermore, studies model fasting responses, diet effects on glycogen, gluconeogenesis, and protein metabolism [54]. Additionally, relationships between metabolites and reactions can be described to estimate the evolution of biochemical pathways during disease progression [40,48]. Glucose-insulin-glucagon interactions, gut absorption, and insulin action can be represented using differential equations [66,68,84]. Single-hormone models, which can use ODEs, describe insulin kinetics and dynamics, as well as carbohydrate absorption, enabling the generation of virtual patients with varying insulin sensitivity. Dual-hormone models extend this by incorporating glucagon dynamics and kinetics [84]. The literature models exercise via insulin sensitivity adjustments based on oxygen consumption [84].

#### Simulators and Model Optimization Methods

Process representation often involves metabolic simulators that model key physiological processes to educate patients and personalize treatment plans [37]. The literature shows that the core mechanism of the DT is grounded in mathematical functions fitted to individual patient data. Model parameters can be either global, representing fixed knowledge, or optimized using data-driven or mathematical approaches.

Commonly, disease progression, glucose-meal responses, glucose-insulin dynamics, and hypoglycemia risk are simulated. For instance, a metabolic network can be modeled using a generalized stoichiometry matrix, where linear systems can represent biochemical reaction fluxes, while scalar variables can identify time-dependent fluxes and metabolic changes [40,48]. Meal responses can be simulated, incorporating knowledge of human metabolism derived from clinical studies, to describe personalized responses to meal compositions and decrease the risk of diabetes onset [54]. Additionally, deterministic models based on linear discrete-time systems are proposed to simulate glucose-insulin dynamics. Such a model would require 4 interconnected modules representing the gastrointestinal tract, subcutaneous tissue, liver, and metabolism, and should also account for daytime insulin and glucose sensitivity [67,93]. Furthermore, a global ranking and collinearity analysis can be used to construct compact matrix representations of linear ODEs. After optimizing the main parameters and the measurement-error covariance matrix, the patient can be represented by a dynamic mathematical model [37]. Clinical applications would include optimizing insulin doses and diets using the virtual patient, time, and the meal plan [67,93]. Another use case is enhanced management of multiple daily insulin injections, especially relevant for children with T1D. Here, the literature simplifies the input parameter complexity of commonly used simulators, such as the UVA/Padova simulator, which models glucose-insulin-carbohydrate interactions [77,94].

Some simulators focus specifically on open-loop insulin optimization, simulating insulin dosing strategies (eg, dual-wave, split-bolus, dual-wave with fixed duration, and standard bolus) while estimating the probability of hypoglycemia [69]. For insulin optimization, glucose levels and response can be simulated iteratively, or multiple statistically described patient representations can be generated and simulated in parallel. These simulators should account for uncertainties in sensor errors, insulin pump delivery, and daily variability in food and insulin responses [5,69,77]. The insulin policy is commonly modeled using stochastic and convex optimization, designed to reduce the average peak BG and minimize constraint violations [69]. Dynamic adaptability is ensured by the model predictive control method, which computes optimal insulin infusion at each time step by minimizing BGL excursions and insulin costs while maintaining target glucose values [23].

Furthermore, simulators based on differential equations or linear affine models can enhance the performance of artificial pancreas (AP) systems. Studies have particularly emphasized the importance of incorporating a set of personalized, time-varying constants and multimodal inputs (eg, daytime, PA, food images, and patient records) [60,65]. Time-varying parameters should be used across all modules, as they describe intrapatient glucose variability [86]. Insulin adjustments should depend on the patient’s daily activity level [60]. Stability can be improved with a scaled error term to penalize implausible chromosome behavior or oscillations [65].

Finally, 1 study presents a metabolic language for a glucose simulator that can model multiweek scenarios. It enables physicians to create and refine physiological models based on medical terminology, which are then translated into mechanistic models. The presented framework initializes the simulated metabolism based on real patients and personalizes it using glucose time series, meal records, and medication data. Such a framework built on differential evolution algorithms can simulate individual real-world scenarios, personalized diet and drug treatments, and support disease management [70].

Parameters are generally grouped into static, which are extracted from validated studies; general, which are optimized worldwide; population-specific, which are tailored to the characteristics of the specific population or demographics; and individual parameters, which are optimized using mathematical or data-driven optimization algorithms [7,54]. Optimization methods can be categorized primarily as linear, nonlinear, statistical, or heuristic approaches [69]. Nonlinear optimization includes convex and quadratic optimization methods [40,48]. Statistical optimization encompasses Bayesian methods, expectation-maximization, semiparametric regression optimization [23], and probabilistic techniques such as Markov chain Monte Carlo [5,77]. Finally, heuristic or metaheuristic approaches include scatter search [7], genetic algorithms, and particle swarm optimization [65,70].

To improve prediction accuracy and prioritize recent glucose values, the literature incorporates a forgetting factor. Further optimization and stability are achieved through slack variables and constraints, such as recommended BGL and insulin infusion limits [23]. Studies also address variability and uncertainty through fuzzification and probabilistic or stochastic elements, since human biological processes are dynamic and less deterministic [67,93]. For instance, a Profile Likelihood method can assign variation to unmeasured factors like stress, exercise, or meal intake [85].

In conclusion, simulation processes are usually represented using linear, nonlinear, or statistical methods. Methods can also be classified as deterministic or stochastic [66]. Most studies adopt hybrid methods, in which nonfixed parameters are optimized and tailored to the data and context. Finally, many studies already integrate several physiological abstractions, including organ, tissue, cellular, and behavioral processes, to model a whole-body system [7,8,24].

### Data-Driven Representation

#### Overview

Across the reviewed literature, data-driven approaches use statistical and ML models to detect patterns, predict anomalies, and assess disease progression. These models support decision-making systems, further enhanced by explainable AI (XAI) [47]. By continuously learning from historical and real-time data, they enable precision medicine through personalized therapy recommendations, refining predictions over time to improve accuracy and adaptability. Furthermore, complex relationships within the data can be uncovered [44]. A key characteristic is the automatic refinement of models with incoming data or user feedback, dynamically selecting the best-fitting model and integrating improved alternatives. This adaptability defines them as closed-loop DTs [5,43].

This section presents use cases of data-driven representations and categorizes existing approaches into disease diagnosis and progression prediction, glucose forecasting and simulation, and lifestyle recommendations. Common use cases and data-driven methods are summarized in Table S4 of Multimedia Appendix 2.

#### Disease Diagnosis and Progression

DTs can be used to estimate the risk of disease onset and progression to diabetes based on data trajectories over time. Notably, the temporal evolution of biomarkers associated with T2D onset has been described using multi-input, multioutput models. For instance, a multivariate Gaussian process model with an autoregressive structure, accounting for interdependencies between outputs, is proposed. It contains input parameters for a specific time window and output parameters for the next time window, enabling the comparison and characterization of biomarker dynamics. Additionally, patients can be categorized into high- and low-risk groups based on their biomarker evolution, enabling individual risk assessment years before onset [95]. If already diagnosed with diabetes, disease progression and the onset of comorbidities can be predicted. Binomial logistic regression models trained on demographics and metabolic fluxes can provide predictive insights into baseline identification and the future onset of complications such as CKD, diabetic retinopathy, and cataract. By incorporating correlation analysis, studies identify key parameters contributing to elevated GMF profiles. The literature further evaluates disease progression by analyzing correlations between health-state distance metrics and patient diagnoses, while distance-based classification methods enable stratification of patients into high- and low-risk groups for increased personalization [40,48]. Usually, regression-based models forecast changes in parameters. In contrast, binary classification can classify changes in value (eg, of at least 5%), leading to lower variance [25]. Moreover, statistical methods such as Kaplan-Meier analysis and Cox proportional-hazard models can estimate progression rates, showing the probability of remaining healthy or the risk of progression of a comorbidity, respectively [40,48].

To diagnose diseases using multitarget binary classification, a hybrid model consisting of ML and neural networks is proposed to predict health care metrics such as peripheral capillary oxygen saturation, body temperature, and diabetes [80]. By including multimodal inputs such as genomics, CGM device data, and wearable data, additional comorbidities can be predicted before onset, and interventions can be tailored. In particular, extending models with neuroimaging can forecast dementia risk, which is correlated with diabetes [96]. Image classification techniques can detect and categorize diabetic retinopathy based on severity (mild, moderate, or severe). Model confidence is increased with an integrated feedback layer, enabling HPs to provide input, facilitating continuous model refinement and improved diagnostic performance [57]. Another proposed use case for diabetes-related DT systems includes chronic wound management, a common complication of T2D, requiring periodic examinations and continuous care. Via image processing and AI models, the concept predicts healing trajectories to assist clinicians in determining treatment plans. Such a framework should support the entire model lifecycle, from creation through experimentation to continuous monitoring and refinement [58].

#### Glucose Forecasting

The main research and application domain is glucose forecasting and hypoglycemia prediction using neural networks and deep learning (DL). Across the literature, glucose values were forecasted using different prediction horizons of short-term (5 to 60 min) or long-term (24 h). For short-term predictions, models based on long short-term memory (LSTM) or its hybrid variants (eg, gated recurrent unit–LSTM and convolutional neural network–LSTM) are most often applied to multisensor inputs or multimodal data [14,23,64]. For daily glucose predictions, models based on artificial neural networks [62] or GANs using recurrent neural networks with a combination of supervised and unsupervised learning to learn temporal dynamics in latent spaces are presented [47,59]. Furthermore, studies apply multilayer perceptron models to predict glucose levels using transformer-based vector encodings of the input data [61].

Besides glucose forecasting, blood peaks can be forecasted with hybrid models that use a CatBoostRegressor for categorical variables, a random forest model to model the nonlinear relationship between meals and glucose, and an LSTM to capture temporal patterns [56]. Thus, DL-based glucose simulators can be generated to predict glucose concentrations for the next day using sensor input, lifestyle behaviors, and the patient’s individual characteristics. Simulators can be used to explore new treatments by modifying conditional inputs and predicting corresponding glucose responses, thereby enhancing decision support and awareness [14,59]. With threshold-based comparison, the forecasted glucose can alert to predictive adverse events, predict the risk for hyperglycemia or hypoglycemia, and the time spent in target ranges [14,61,95]. Moreover, structured TSA is used to detect hyperglycemic and hypoglycemic patterns [23]. Further insights are provided in reviews of different algorithms that do not incorporate a DT framework [51,97,98].

#### Lifestyle Recommendation

Based on predicted glucose data, the literature generates further lifestyle recommendations, augmenting decision support. For instance, data-driven insulin titration policies are proposed based on reinforcement learning. Methods can include the soft actor-critic with entropy-driven reward functions or reward functions based on target glucose levels [14,47,61]. Dosing strategies can be continuously refined and improved using data-driven methods that integrate patient representation, real-time data, and other relevant predictions [61]. Another research field is nutrition and diet suggestions. After analyzing food intake, studies outline unsuitable choices to optimize nutrient balance and prevent over- or underconsumption, such as adding vitamins or proteins [39]. Personalized dietary recommendations can be enhanced by integrating patient profiles, behavioral factors, and DTs of food generated through mathematical modeling and simulation of meal components. Notably, ML models are used to identify diet patterns, forecast optimal food choices, and reduce diet-related disease risks [63]. Moreover, a KG embedded with logic rules that provide the patient’s management plan, allergies, and diet preferences can reason whether a meal meets the requirements [47]. Methods can also predict weight with neural networks and be optimized to target weight with particle swarm optimization algorithms [62]. The predicted blood peaks can personalize diet recommendations using a multiobjective optimization strategy that considers glucose profiles, nutrition, activity, sleep, and stress, to minimize glycemic variability while maximizing nutritional quality [56]. Further use cases include drug recommendations, where the disease is first identified from symptoms and drugs are then suggested, aligned with those symptoms using ML models [39].

#### XAI

Lastly, XAI is introduced as a submodule. XAI can explain glucose trajectories or provide insights into the parameters that lead to adverse events. For instance, an XGBoost (Extreme Gradient Boosting) classifier, serving as a base learner, enhanced with the LIME (locally interpretable model-agnostic explanations) tool, is used to explain predicted adverse events to improve self-management of diabetes [23]. Furthermore, risk factors for the onset or progression of diabetes can be prevented with personalized countermeasures. Counterfactual explanations, which are part of local XAI methods, predict outcomes based on modified input parameters to explain why the model made a specific decision, identifying necessary changes leading to the optimal outcome. The counterfactual explanations can be assessed using biomarkers extracted from EHRs and clinician survey responses [8,99]. Another proposed method is based on a Gradient-Weighted Class Activation Mapping model. It identifies key factors contributing to adverse events and allows iterative adjustments to test intervention impacts virtually [14]. Finally, after generating predictions and recommendations, large language models based on a generative pretrained transformer can provide explanations for their predictions and suggestions in a user-friendly report [5].

### DT Approaches

#### Overview

This section highlights promising studies that can be defined as preliminary DTs or implemented DT approaches, whereas DT-enabling components are not considered. A detailed analysis of multilevel representation frameworks is provided in Table 1. Table S1 in Multimedia Appendix 2 summarizes all studies that present themselves as DTs, including single representations, but excluding concepts and simulators that do not provide new insights or have been previously discussed. Most studies do not propose holistic frameworks and cannot tailor their model to the patient. In particular, the generation of patient profiles and the representation of patients as virtual replicas are frequently neglected. Data representation accounts for only 25% (7/28), which requires data organization and structured management that goes beyond basic data collection and preprocessing. In addition, only 5 studies include model refinement for continuous adaptation and improvement. The distribution of process- and data-driven representations is balanced, with 64% (18/28) and 61% (17/28), respectively. Analyzing the individual studies, single-level abstractions are addressed in 64% (18/28) of studies, with the majority using methods for process representation, followed by data-driven approaches, whereas data-only representations are not found. Two-level abstractions, primarily integrating data and data-driven representations, account for 21% (6/28) of studies, while combinations of data and process representations are less common. In this context, mechanistic models optimized using data-driven techniques are not classified as 2-level representations. Finally, 3-level representations are covered by 14% (4/28) of the studies.

**Table 1.** Multilevel digital twin approaches for diabetes.

Study	Target	Representation			Clinical use case		Classification	
Vaskovsky et al, 2020 [55]	Pre-D^a^	Data + data-driven R^b^			Meal recommendation		Preliminary DT^c^	
Keshary et al, 2022 [60]	T2D^d^	Process + data-driven R			Insulin management		Preliminary DT as offline simulator	
Batagov et al, 2023 [48]	T2D	Three level R			T2D management		DT as offline simulator	
Cappon et al, 2023 [5]	Pediatric T1D^e^	Process [77]+ data-driven R			Insulin management, telemonitoring		DT as online simulator	
Lee et al, 2023 [9]	T1D, T2D	Process [100,101]+ data-driven R (concept)			Comorbidity, insulin, and lifestyle management		Preliminary DT as online platform	
Paglialonga et al, 2023 [8]	Pre-T2D	Process [88,90-92]+ data-driven R			Prevent T2D onset		Preliminary DT	
Thamotharan et al, 2023 [23]	Older adult T2D	Three level R			Insulin management		Educative DT	
Wang et al, 2023 [61]	T2D	Three level R			Insulin management for AP^f^		Preliminary online DT	
Rad et al, 2024 [47]	T1D, T2D	Data + process (concept) + data-driven R			Glucose management		Preliminary online DT	
Shamanna et al, 2024 [56]	T2D	Data + data-driven R			Medication and meal management		Preliminary online DT	
Surian et al, 2024 [40]	T2D	Three level R			CKD^g^ risk monitoring		DT as offline simulator	
Zhang et al, 2024 [25]	T2D	Process (concept)+ data-driven R			T2D risk monitoring		Preliminary DT framework	

a Pre-D: prediabetes.

b R: representation.

c DT: digital twin.

d T2D: type 2 diabetes.

e T1D: type 1 diabetes.

f AP: artificial pancreas.

g CKD: chronic kidney disease.

According to Table S1 in Multimedia Appendix 2, simulators are the most common components. Simulating processes and trajectories is essential to forecasting events and adjusting treatments. Nevertheless, they do not qualify as full DTs on their own. Data are usually collected in cloud storage and visualized through smartphone apps. However, these data are often not further processed, structured, and modeled for knowledge representation or AI-ready formats. Key use cases of all the retrieved literature include virtual trial simulators, which refine therapy parameters by predicting long-term outcomes, generating synthetic data, forecasting glucose trajectories or insulin responses, and aiding in selecting a healthy lifestyle. Additionally, DT models are frequently used to predict disease onset and progression. Table 1 reveals that, in multilevel DT approaches, insulin optimization is usually addressed.

#### Two-Level Approaches

Proposed 2-level architectures were usually classified as intermediate DTs because they do not fully implement and integrate multilevel representations. These approaches differ in maturity and clinical scope across the literature. Paglialonga et al [8] use complementary process- and data-driven abstractions to enhance functionality for a specific clinical use case. They extend the process representation toward a multiorgan, metaflammatory system. Data-driven approaches estimate the risk of T2D onset using personal data, forecast progression to T2D onset, and define personalized recommendations [8,95,99]. The process representation integrates a gastrointestinal tract module, a PA model, and hormonal responses to evaluate the impact of the individual’s metaflammatory status on developing T2D risk [8]. This work presents a framework in which subcomponents have been tested with real clinical data but not integrated into a single meta-architecture [73,95]. In contrast, Lee et al [9] integrate both representations for separate applications. A cloud-based data collection platform facilitates communication between patients and HPs, enabling feedback, titration, and education [9]. However, in the reported architecture, this layer primarily functions as a storage and data-sharing platform rather than as a structured virtual representation of the patient. As no explicit semantic, relational, or patient-specific data model is described, this layer was not classified as a data representation within our framework. They described a stochastic simulation framework that models diabetes-related conditions with separate transition probabilities and management strategies for T1D and T2D, which is notable because most reviewed approaches focus on a single diabetes subtype. The process representation estimates interactions among diabetes-related complications at the individual-patient level [100,101]. In parallel, a T1D-focused component uses wearable and personal data (eg, CGM, insulin pump, food-tag, and activity) to support insulin and lifestyle management [9]. Besides the data collection platform, the proposed architecture remains largely conceptual and is not presented as a fully implemented or clinically validated system. Furthermore, 1 study considers personalized multiple daily insulin adjustment in children with T1D. Cappon et al [5] model the glucose-insulin metabolism and generate therapy suggestions. The process representation involves twinning, simulation, and recommendation procedures using an open-source methodology [77] to represent a person’s physiology. They present a data layer focused on AI readiness, including data preprocessing, labeling, and context extraction, but do not report a patient representation layer, which is why we did not classify their method as a full data representation. The system integrates all modules to provide interaction with data and HPs. Similarly, the data-driven representation is not fully developed and is used only as an LLM to generate suggestions and provide user-friendly text for HPs and users [5]. However, as heterogeneous data is used and the system aims for personalized models with multiple subcomponents, it was not considered a single simulation framework. This system comprises partly implemented components, but most are not validated with real clinical data, and the meta-system remains a framework. Likewise, Keshary et al [60] present a personalized insulin optimization but for AP systems in geriatric patients with T2D. The system combines a patient simulator with data-driven meal recognition from images and activity-aware insulin optimization. Individual components were evaluated in silico, while the patient simulator and insulin optimization were further computed using real clinical data. Although the study is not explicitly framed as a DT system, it was included as a DT-related prototype because it combines multimedia data collection, patient-specific simulation, and individualized insulin optimization [60].

Data and data-driven representations typically structure information using ontologies and preprocess data for ML submodules. In this context, Zhang et al [25] develop a T2D-based DT model integrating demographic, clinical, and multiomic data structured as KGs, which should provide data for mechanistic and data-driven models. The KG maps biomedical relationships, identifies unknown parameters, and interprets predictions. Additionally, clinical trajectories and progression to CKD are predicted using ML. The models are validated with real clinical data. However, as the data structures provide domain-specific information rather than representing patient data, they do not yet qualify as a comprehensive data representation. Moreover, the process representation is only mentioned as a conceptual framework. In contrast, Rad et al [47] develop a hierarchical, top-down, personalized ontology tailored to diabetes (types 1 and 2) and use ML models with continuous refinement to uncover complex patterns, disease risk factors, and health trajectories. Proposed applications include personalized insulin adjustments to minimize hypoglycemia risk, 24-hour glucose forecasting, KG exploration, and logic-driven personalized meal recommendations. Nevertheless, this approach lacks a process representation. Furthermore, the predictive modules are only validated with virtual data, while the integrative meta-system was not fully implemented. Using the same representation levels, Vaskovsky et al [55] and Vaskovsky and Chvanova [74] differ in the data format and use case. They present a conceptual framework for personalized nutrition targeting individuals with a genetic predisposition to diabetes based on tabular data. The system leverages a nutrition knowledge base with machine-rule-based logical inferences, structured according to HL7 standards. First, the framework creates a twin based on individual data (eg, EHR data, taste preferences, and individual goals and restrictions), and synthetic patient groups are computed. Second, the twin of the food product is created from the given meal components. Third, an inference machine analyzes the effects of food items on digestion, absorption, and metabolism. Finally, personalized diets are recommended by modeling interactions among genetics, microbiomes, immunity, and metabolism, influenced by diet and activity.

Lastly, Shamanna et al [56] propose a fully implemented system that generates patient profiles and uses AI for prediction. In contrast to Vaskovsky et al [55,74], Shamanna et al [56] propose a DT system for T2D comprising a data management, prediction, and recommendation layer, with the data organization unspecified. A personalized metabolic model is created using wearable data, dietary logs, and biometric inputs. The data-driven representation consists of rule-based expert systems and AI models using vital signs, bloodwork, nutrition, and demographics [28,81]. Similarly, the prediction model evaluates the impact of a meal. Glucose peaks are predicted and penalized for medication use to account for artificially reduced levels. Whereas Paglialonga et al [8] use redundancy by combining process- and data-driven abstractions, this system introduces redundancy within the data-driven layer for glucose-response prediction. Personalized decision support is provided for medication, diet, and exercise [28,56,81]. A feedback layer enables continuous refinement, adapting to new data, trends, and cohort-based models [56]. Finally, the telemonitoring module enables HPs and coaches to monitor progress, manage medication, and provide support. This system has already been used in randomized controlled trials [28,56,81].

In general, the approaches remain conceptual frameworks, are mostly tested sparsely or in silico, and are neither fully implemented nor ready for clinical trials or clinical use cases, except for the system of Shamanna et al [56].

#### Three-Level Approaches

For three-layered representations, the included literature reports implemented (retrospective clinical) proof-of-concept studies with clinical data, whereas no included architecture was used in clinical trials. Moreover, across studies, the role of the representation layer differs. For instance, Wang et al [61] create an insulin policy model but do not incorporate biological processes. They present a DT model for dynamic insulin dosing for patients with T2D. A patient model encodes patient-specific data from EHRs, historical data, and continuous data using NLP and a transformer, which tracks evolving states, generates transitions, and estimates rewards from past trajectories. The module computes hidden states and internal patient conditions, and data-driven methods forecast glucose levels, target-range compliance, and the following patient state. Interacting with the patient model, the policy model optimizes the insulin dose based on the current state [61]. While Thamotharan et al [23] rely on sparse ML models as additional or complementary components rather than as the main simulator, they also focus on personalized insulin infusion. They present a DT adapted to older adult patients with T2D using contextual and geriatric data. The module assesses key indicators such as time in range, adverse events, and model accuracy. Mechanistic equations describe glucose-insulin interactions and are optimized to patient-specific parameters. Likewise, personalized insulin infusion is supported through recursive refinement of glucose, insulin, and carbohydrate dynamics. Furthermore, glucose is forecasted with data-driven models. In contrast to Wang et al [61], their system is expanded to multiple subcomponents. The prediction module integrates glucose forecasting and structured TSA. A matrix profile detects recurring motifs, anomalies, and inter- and intrapatient patterns affecting glucose trajectories. Then, anomalies, sensor errors, and influential activity patterns are detected. It further integrates XAI to interpret hyper- and hypoglycemic events. Finally, physicians’ recommendations and patient conditions are integrated for improved diabetes management [23]. Similarly, Surian et al and Batagov et al [40,48] are classified as mechanistic physiological-biological approaches supported by data-driven methods. Both systems rely on the same underlying representations and principles, but extend the DT to different use cases. The DT is based on a metabolic flux network, in which metabolic reactions and pathways are represented through GMF analysis. The model is initialized with patient-specific metabolite and physiological data, while metabolic states are formalized through a stoichiometric matrix fitted to clinical data. This enables personalized prediction of metabolic states and disease trajectories, as well as the identification of comorbidity-associated patterns. Health state distances measure disease progression and identify patient subgroups at high or low risk. In addition, data-driven methods incorporating demographic and metabolic flux data are used to predict disease onset and progression, quantifying risk using health-state distance metrics [48]. While Batagov et al [48] investigate progression to cataract and retinopathy within 3 years, Surian et al [40] predict CKD over 3, 5, and 10 years, classifying patients into high-, moderate-, and low-risk groups. They enhance the model with nutritional and respiratory data and use it to model microvascular complications leading to CKD, incorporating medication effects. Here, mathematical models structure the patient’s state and changes, while data-driven methods forecast disease risk.

In summary, 3-level representations aim either to optimize insulin or to predict disease onset and progression, while emphasizing personalization and incorporating context and person-specific information.

### Clinical Use Cases of Diabetes-Related DT Systems

While most studies use the same core methods and similar inputs, the clinical use cases differ. Common identified application areas are insulin therapy optimization [5,23,27,47,60,61], exercise-aware decision support [23,24,60], diet and behavioral recommendation [5,7,8,23,27,47,54-56], disease risk, onset, or progression simulation (eg, CVDs, neuropathy, retinopathy, and nephropathy) [7-9,25,40,48,57], medication monitoring [56], telemonitoring [5,9], and patient education [8,23,65,68,71]. Insulin-related use cases primarily address T1D [5,27,47], older adult patients with T2D or insulin-treated T2D [7,23,60,61], and include insulin dose adjustment, personalized insulin infusion, and AP optimization for single- or dual-hormone systems. Exercise-aware decision-support systems aim to reduce hypoglycemia risk during activity by suggesting insulin dose adjustments, eating snacks, postponing or adjusting exercise [24]. For T2D and type 2-prediabetes, DT-related systems more frequently predict disease onset, disease progression, and comorbidity risk, or provide lifestyle recommendations [8,40,48,56]. Some systems also focus on patient awareness, self-control, telemonitoring, and clinician-patient communication via data platforms [5,9]. Finally, educational and gamification-based systems represent a distinct use case. These aim to improve patient awareness, self-management, and understanding of glucose dynamics by allowing users to observe the simulated effects of behavior [68,71]. Such systems are particularly relevant for children [71].

## Discussion

### Overview

This study presented the current state of research on DTs for improved diabetes management. The approached use cases of diabetes-related DTs were systematically synthesized, providing an overview of the individual components. This section addresses the initial research questions and discusses identified research gaps.

### Characteristics of Diabetes Related DTs

Diabetes-related DTs are characterized by the integration of patient-specific data, diabetes-relevant physiological processes, and adaptive, data-driven methods. Across the reviewed literature, data representations primarily comprise heterogeneous patient information, including continuous physiological parameters, EHRs, comorbidities, medications, dietary restrictions, FH, and lifestyle data. Process representations model diabetes-related mechanisms, particularly glucose regulation, metabolism, insulin action, meal responses, PA, and disease progression. Data-driven representations use static, categorical, and time-series data to predict glucose levels, disease onset and progression, optimize insulin, or provide decision support. Overall, the main characteristic of diabetes-related DT systems is the degree to which their representations are individualized and integrated. Most reviewed systems implemented only selected parts or components, such as simulators, and did not represent the patient as a virtual data component. Additionally, no dynamic, bidirectional link between the human and their virtual replica [38] was proposed in diabetes-related studies. Hence, many studies were characterized as DT-enabling components or intermediate DT architectures rather than complete, closed-loop, or holistic diabetes-related DTs.

### Data Layer

Diabetes-specific DTs integrate complex and heterogeneous data sources, including meal intake (particularly carbohydrate composition), insulin dose, demographic characteristics, and physiological parameters such as CGM data, finger-prick glucose levels, and PA. Additionally, heart rate and blood pressure provide insights into comorbidities. Data preprocessing techniques, such as automated meal intake estimation or activity specification, are used to reduce the burden of manual data entry. Data storage is predominantly cloud-based, leveraging the IoMT infrastructure. Some approaches incorporate semantics, KGs, and ontologies to extract relationships and enhance explainability, while most systems integrate mobile apps to facilitate data visualization, user feedback, and real-time data logging.

### Model Layer

Primary use cases include glucose-level forecasting with DL and simulation using mechanistic models that represent metabolic responses to meals, insulin, and PA. A few studies also incorporate modules for drug response and inflammation. Physiological modeling occurs at multiple scales, ranging from tissue and cellular levels to organ-level representations, including the liver, gastrointestinal system, and renal function. Insulin dose adjustments and personalized recommendations for nutrition, medication, and exercise are made using mathematical or data-driven models. Additionally, predictive models assess disease progression and the onset of diabetes-related comorbidities to provide decision support. Lastly, some implementations incorporate educational modules that enhance explainability by evaluating the impact of features on disease dynamics. Both approaches can provide explainability, while data-driven methods are guided by XAI to enable transparent recommendations. Both methods are interrelated, augment each other, and can be used as hybrid architectures for similar use cases. Mathematical models are based on known mechanistic and physical relations, which can be formalized and optimized using the available data. In contrast, data-driven representations are defined as the individualization component, as they depend solely on the data to identify hidden patterns, rather than on fixed model parameters [38].

### Patient Representation

Patient representation commonly involves vectorization techniques, patient-specific matrices, KG representations, and ODEs. Optimization strategies encompass linear, nonlinear, statistical, heuristic, and stochastic methods. While 3-level representations that integrate data, process, and data-driven models remain an emerging area of research, 2-level representations, such as data representation combined with data-driven models or process models augmented with data-driven techniques, are more commonly investigated. Despite significant advancements, further research is needed to develop holistic personalized DTs that effectively integrate clinical, biological, and behavioral data to optimize personalized diabetes management.

### Clinical Implications

The reviewed literature suggests that diabetes DT architectures require adaptation across patient populations and diabetes subtypes. Most studies distinguish between T1D, T2D, or type 2-prediabetes, and some approaches further adapt models to diabetes subtypes [65,85,100,101]. Relatively few studies focus on specific populations, such as older adult patients with T2D or children with T1D. This indicates that future diabetes DTs should not be designed only for isolated populations but should account for age and disease subtypes. Additionally, the generalization of models and systems is not evaluated. Pediatric DTs may require age-specific metabolic representations, safety constraints, and education-oriented recommendations, whereas DTs for older adult patients should incorporate comorbidities, reduced activity, and dietary limitations. Another underexplored challenge is model bias and fairness, which pose risks of algorithmic discrimination against populations underrepresented in the training data [102], thereby limiting equal access to the benefits of the technology.

DT-related systems most commonly focus on glucose prediction, personalized insulin support, diet and lifestyle recommendations, and disease onset assessment. However, only 2 systems demonstrated clinical real-world applications. Most approaches remain conceptual, are evaluated only in silico or with small datasets, and are not validated with external datasets. Notably, 3-level representations were generally validated using real clinical data. Overall, the reviewed evidence indicates that diabetes-related DTs have strong potential for individualized care, but most systems remain at the prototype or proof-of-concept stage rather than being ready for routine clinical implementation. Beyond technical capability, reliable implementation depends on legal and ethical considerations that are not considered by diabetes-related studies. For instance, DTs should be equally accessible, but are costly to develop and maintain, limiting access for resource-constrained hospitals and institutions [102,103]. Moreover, data ownership and governance remain unresolved, particularly in multisource, multicomponent DT systems [102,103].

### Research Gaps and Limitations

Notably, there are some limitations. Figure 5 summarizes the main findings and presents challenges. A major research gap is the absence of a unified methodological framework or standard for HDTs, integrating key components. Additionally, a lack of common definitions has led to DTs being equated with simulators or isolated DT-enabling components, although these represent only partial elements of a more comprehensive DT architecture. In this review, 3-level systems represented the highest maturity group because they combined individualized data, process representation, and data-driven prediction or optimization. Nevertheless, most reviewed approaches remain preliminary foundations. In medicine, DTs should serve as comprehensive knowledge bases that incorporate individual history and personal characteristics. They should go beyond simulating or forecasting isolated events while integrating interrelated submodules.

**Figure 5.**
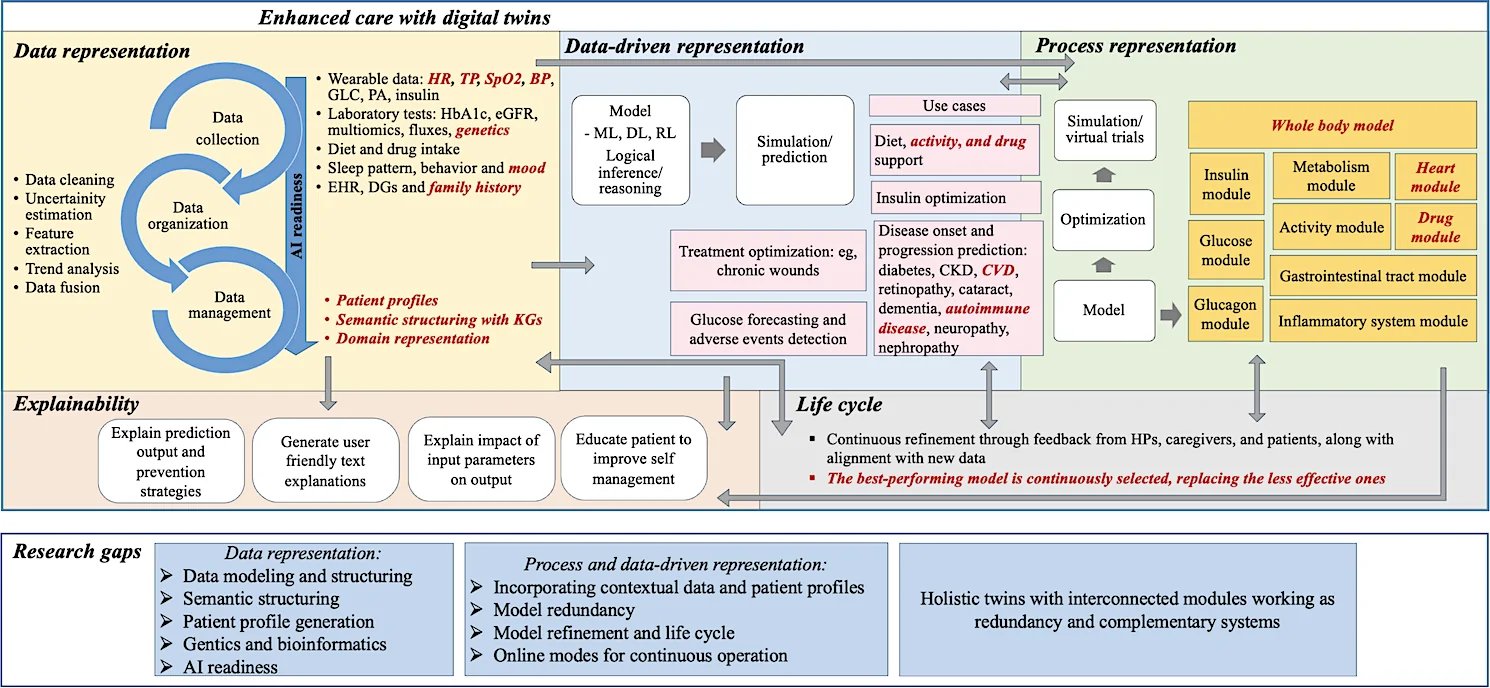
Enhanced diabetes management with DTs and identified research gaps (1) Describes the diabetes digital twin framework, highlighting underexplored processes in red. Data is collected, organized, and structured within the data representation layer to ensure AI readiness. This layer interacts unidirectionally with the process and data-driven representations, and explanation layer. The process representation models biological processes, while the data-driven layer applies AI models to predict events and states. These 2 layers are bidirectionally connected and have a unidirectional connection to the explanation layer. All representations bidirectionally interact with the life cycle layer and are refined continuously. (2) Summarizes main research gaps. BP: blood pressure; CKD: chronic kidney disease; CVD: cardiovascular disease; DG: demographics; DL: deep learning; eGFR: estimated glomerular filtration rate; EHR: electronic health record; GLC: glucose; HbA_1c_: hemoglobin A_1c_; HP: health care providers; HR: heart rate; KG: knowledge graph; ML: machine learning; PA: physical activity; RL: reinforcement learning; SpO_2_: peripheral capillary oxygen saturation; TP: temperature.

Furthermore, the absence of a standardized approach for data integration and preparation complicates the use of multisource heterogeneous data. Clinical data and EHR data integration remain challenging due to structural variability, privacy concerns, and liability issues [43,44]. Additionally, the diversity of EHR platforms, inconsistencies in medical terminology, and gaps in critical patient information further obstruct seamless interoperability, as reported by Lee et al [9]. Particularly, standards such as HL7 and Fast Healthcare Interoperability Resources should be adopted [42,44], yet only 2 studies have incorporated them [47,74]. Additionally, DTs should not be restricted to specific systems or devices and require interoperability and standardization across heterogeneous digital ecosystems, including medical platforms and devices [102,103]. Notably, clinical adoption of DT technology depends on the seamless integration into existing workflows without increasing the workload of clinicians and personnel. Consequently, establishing standardized definitions, terminology, and sensor- and system-agnostic frameworks is essential for advancing the field, ensuring the sustainability of DTs, and enabling equal access.

At the single-representation level, data representation of patient information remains largely overlooked, despite its importance in developing personalized DTs that go beyond mere simulation. This limitation is also observed in the broader study of DTs not tailored to diabetes [104]. It is primarily limited to data collection via smartphones, edge computing, sensors, and cloud storage, with few studies incorporating EHRs or extracting data using NLP. Most rely on raw datasets or manually preprocessed data, and model performance is often constrained by sensor brands, limiting generalizability [86]. AI-ready data and handling variations in data collection intervals remain unaddressed, despite their strong correlation with AI model performance [75]. Further, validation across multiple datasets is rarely conducted. Despite the availability of multiple sensor sources, applications are not adapted to stream data, as most studies rely on precollected datasets rather than real-time processing, limiting the maturity of real-time applications [102]. Preprocessing and data transformation remain underdeveloped, limiting AI readiness for submodules. Proper structuring and management are essential for data fusion, pattern identification, profiling, and the extraction of causal relationships or pathways. Preprocessing efforts mainly focus on data imputation and cleaning, while some automate the extraction of meal types, activities, and lifestyle factors to reduce manual input.

Data management is another critical gap, with only 5 studies developing KGs, ontologies, vector representations, or semantic integration, and just 2 studies mentioning data storage using SQL tables. It is recommended to leverage existing ontologies aligned with medical standards. For instance, El-Sappagh et al [105] propose an ontology for diabetes management that integrates IoT sensor data [106]. They cover diabetes diagnosis, drug interactions, and diabetes-related complications. Such an ontology can serve as a foundational structure for prediction or simulation models, enhancing model explainability and potentially advancing performance.

A significant research gap exists in bioinformatics, particularly in integrating genetics and FH, which are crucial for T1D and associated immunological comorbidities. This is relevant because factors such as gut microbiome composition and genetic variation play a crucial role in glucose and weight management [62]. Integration of genomic, epigenomic, metabolomic, proteomic, and microbiome data is mostly conceptual or suggested for future work [9,56]. Advancements in noninvasive sensors capable of measuring vital parameters and estimating blood components could further simplify data collection while offering deeper insights into real-time physiological changes. Thus, a virtual data representation for the individual patient has not been effectively implemented, with semantics remaining underexplored and key contextual features yet to be integrated.

In addition, the reviewed literature did not focus on ethical and legal issues related to data ownership, governance, and privacy [102,103]. Currently, it is unclear whether data belong to producers, users, or clinics, whereas many devices give users only limited control [102]. One emerging but not fully developed solution is the Solid personal data pod, which aggregates data from various sources and stores it on patient-selected servers or personal devices [107]. Pods can assign data ownership only to patients and provide them with full control over their data [103,104]. Data and device security challenges are also not considered, which makes the proposed approaches less mature for clinical workflow integration. Only 1 study described using GANs to anonymize data, preserving privacy [39]. However, research in other domains has proposed additional methods, including blockchain and federated learning. Blockchain supports data integrity, transparency, and consistency, while federated learning enables decentralized model training by sharing only model updates and not the actual data [103]. These issues and challenges should be further explored, and effective solutions should be applied to the diabetes domain before introducing the DTs into clinical practice.

Process representation typically uses mechanistic, probabilistic, or mathematical models to simulate biological, biochemical, and physiological processes within the human system. Most studies focus on creating simulators that represent metabolism, particularly the glucose-insulin-meal (exercise) dynamics. Proposed submodules include representations of renal, liver, and gastrointestinal functions to model glucose responses. Moreover, comorbidities such as CKD and eye-related conditions are modeled. However, additional submodules should be considered for diabetes-related complications such as vascular and autoimmune diseases. For instance, glucose fluctuations have been reported to correlate with ECG and heart rate, yet no existing glucose simulator can predict heart-related values. Additionally, PA is often underrepresented in metabolism models, despite its critical impact on glucose values. In particular, exercise recommendations play a critical role in preventing the onset and progression of disease [8,108].

While existing models are optimized based on age and gender, they do not differentiate between children, adults, and older adults, even though biological processes may vary across these groups. Furthermore, for accurate glucose simulation, incorporating CGM as a submodule is essential. These modules should be designed to function universally, rather than being limited to specific sensor brands. This level of representation is well-suited for capturing mechanistic process knowledge and identifying explainable factors for parameter adjustments. While these models can be fitted to individual values using efficient optimization techniques, they are limited in their ability to integrate contextual and historical data, which may significantly influence metabolism.

In contrast, data-driven methods leverage all available data and can integrate various data architectures to model and predict individual trajectories or event risks. Most approaches focus on glucose forecasting or simulation, with limited trend analysis. Particularly for diabetes management, submodules predicting hypo- or hyperglycemic states or extreme conditions are important. Comorbidity submodules usually cover conditions such as CKD, retinopathy, cataracts, but heart diseases and hypertension have not been explored. A promising potential submodule for data-driven approaches could involve forecasting an individual’s remaining lifespan under an unchanged lifestyle, similar to the approach introduced by Lakshmi et al [109] or applied in aircraft maintenance [110].

Moreover, future studies should extend personalization beyond individualized physiological models, simulations, and predictions that rely solely on EHRs, personal data, and wearable-derived time series. Medical care has shifted toward person-centric therapies, characterized by greater patient involvement in decision-making and treatment planning [111]. This requires integrating patient-specific goals, burdens, and preferences into model personalization to support accurate and timely prediction and intervention [47]. Hence, medical DTs should integrate a flexible, patient-centric layer that continuously adapts to the patient’s well-being, quality of life, and context. In particular, diabetes-related DTs should learn about patients’ goals, such as completing 2 hours of daily PA and avoiding nocturnal hypoglycemia. The model should also account for constraints, such as following a gluten-free diet or having long meetings at work that lead to postponed meal times, as well as preferences, such as declining to use an insulin pump.

While mechanistic models are often used to address the lack of explainability in AI models, and data-driven approaches compensate for the limited individualization of mathematical models, the full integration of both remains underexplored. This research gap leaves opportunities for further advancement in model explainability and refinement. Therefore, both representation levels should be implemented as complementary submodules that introduce redundancy, as demonstrated by Thamotharan et al [23] and Batagov et al [48]. Conclusively, DTs that fully incorporate and connect hybrid mechanistic and AI models remain underexplored, representing a gap in this field. Other domains, for instance, use NeuralODEs, physics-informed neural networks, hybrid state space models, or constraint-based learning [72,112,113].

In addition, a virtual twin should remain available in the cloud even when disconnected from its physical counterpart [114]. However, most proposed approaches operate in an offline mode, limiting continuous monitoring.

Finally, model redundancy and refinement are critical for dynamic learning and modeling, ensuring that the best-fit model is consistently selected. Calibration and automatic model refinement are essential to create a DT that accurately represents a patient’s life cycle. Still, this aspect is rarely addressed, while studies emphasize its importance [34]. Prediction is usually based on simple models with limited refinement. Rivera et al [34] propose an evolving DT that continuously integrates additional modules and data as the disease progresses. Each DT instance would consist of a dataset mapped to an automatically generated schema and behavioral models that continuously adapt to new conditions. This dynamic approach enables continuous model evolution and more accurate, personalized predictions.

In conclusion, the reviewed studies show promising progress in diabetes-related DTs. Mechanistic, physiological, personalized, and predictive components are already well represented, indicating that multiple use cases can be implemented with the present data. In contrast, for reliable integration in clinical workflow, data integration, person-centric implementation, real-time processing, and legal, ethical, privacy, and security issues should be further explored. Future work should focus on integrating these components into mature, holistic frameworks. Combining multimodal wearables, automated insulin delivery systems, EHRs, patient-reported outcomes, and contextual data could enable more comprehensive and adaptive patient representations.

Limitations of this review include that synonyms and search terms such as “in silico,” “physiological model,” “personalized model,” “artificial pancreas,” “glucose-insulin model,” or “metabolic simulator” were not included in the search string, which could have led to missed subcomponents relevant to holistic DT systems. However, a comprehensive review of these systems was out of scope for this review.

### Conclusions

In conclusion, the development of holistic DTs for diabetes should encompass data, processes, and data-driven representations, thereby creating a comprehensive knowledge base for individual patients. This integration enables AI-ready data processing, simulates biological processes, and enhances predictive analytics. Ideally, process- and data-driven modules should be implemented in complementary ways, introducing redundancy to improve efficiency. In addition, an adaptive refinement layer that continuously updates models and autonomously selects the most suitable approaches is essential to enhance accuracy and personalization. DTs for diabetes are still in the early stages of research, with significant interest beginning in 2023. However, most existing studies focus on preliminary metabolism simulations and predictive submodules, which are often not interconnected. Few studies have focused on fully integrated 3-level representations. Research gaps include the lack of standardized frameworks for human DTs, as well as for data aggregation and integration. Furthermore, architectures that adapt to diabetes type and age remain underexplored. Data representation requires further attention, particularly when developing suitable data architectures and advancing knowledge extraction and feature engineering for submodules and reasoning. Additionally, only a limited number of studies have incorporated historical and contextual data extracted from EHRs, whereas genomic, laboratory, and family medical history data remain largely unexplored. Mechanistic models have been extensively developed and refined, and various optimization techniques have been proposed to enhance model individualization, whereas submodules for exercise, inflammation, and drug use have been less explored. Predictive modules encompass disease detection, glucose forecasting, lifestyle recommendations, and XAI. However, key comorbidities, such as CVDs, are still underrepresented in current research. Addressing these gaps through interdisciplinary approaches and standardized methodologies will be crucial for advancing DT technology in diabetes care.

## Supplementary material

10.2196/91729 Multimedia Appendix 1 Search Strategy for the Individual Databases.

10.2196/91729 Multimedia Appendix 2 Tables presenting (1) diabetes-related digital twin-enabling components, intermediate digital architectures, and digital twin systems; (2) the mechanistic processes and their outcome; (3) data-driven use cases, their model outcomes, and the approaches used; and (4) the collected data of included studies.

10.2196/91729 Checklist 1 PRISMA-ScR Checklist.

## Abbreviations

AP: artificial pancreas
BGL: blood glucose level
CGM: continuous glucose monitoring
CKD: chronic kidney disease
CVD: cardiovascular disease
DL: deep learning
DT: digital twin
EHR: electronic health record
FH: family history
GAN: generative adversarial network
GMF: generalized metabolic flux
HDT: human digital twin
HL7: Health Level Seven
HP: health care professional
IoMT: Internet of Medical Things
IoT: Internet of Things
KG: knowledge graph
LIME: locally interpretable model-agnostic explanations
LSTM: long short-term memory
ML: machine learning
ODE: ordinary differential equation
PA: physical activity
PRISMA: Preferred Reporting Items for Systematic Reviews and Meta-Analyses
PRISMA-ScR: Preferred Reporting Items for Systematic Reviews and Meta-Analyses extension for Scoping Reviews
T1D: type 1 diabetes
T2D: type 2 diabetes
TSA: time-series analysis
VGG-16: Visual Geometry Group 16 Layer
XAI: explainable AI
XGBoost: Extreme Gradient Boosting
