# Supplementary material for: Data, Process, and Data-Driven Representations of Digital Twins in Diabetes: Scoping Review
Source: JMIR Diabetes. 2026 Oct 7;11:e91729. doi: 10.2196/91729 (PMC13646445; doi:10.2196/91729)
Supplement: Supplementary file 2 [file diabetes-v11-e91729-s001.docx]

**Table S1**. Summary of the search strategy for the individual databases.

| Database | Search string | Filters | Date | Search results |
| --- | --- | --- | --- | --- |
| Google Scholar | (("virtual representation") OR ("digital twin")) AND ("diabetes" OR "metabolic" OR "nutrition" OR "exercise") | Publication date ≥ 2010 | August 2024 | 23 |
| IEEE Xplore | (("virtual representation") OR ("digital twin")) AND ("diabetes" OR "metabolic" OR "nutrition" OR "exercise") | No filters used | August 2024 | 52 |
| PubMed | (("virtual representation") OR ("digital twin")) AND ("diabetes" OR "metabolic" OR "nutrition" OR "exercise") | No filters used | August 2024 | 44 |
| Scopus | (("virtual representation") OR ("digital twin")) AND ("diabetes" OR "metabolic" OR "nutrition" OR "exercise") | Article type: articles, conference papers, and book chapters  Language: English and German  Publication date ≥ 2010 | August 2024 | 160 |
