# Supplementary material for: Data, Process, and Data-Driven Representations of Digital Twins in Diabetes: Scoping Review
Source: JMIR Diabetes. 2026 Oct 7;11:e91729. doi: 10.2196/91729 (PMC13646445; doi:10.2196/91729)
Supplement: Supplementary file 3 [file diabetes-v11-e91729-s002.docx]

**Table S1.** Diabetes-related digital twin-enabling components, intermediate digital twin architectures, and digital twin systems.

| Study | Target | Data R. | Process R. | Data-driven R. | Use case |
| --- | --- | --- | --- | --- | --- |
| Goodwin et al., 2019 [69] (J) | T1D | N/A | GLC-I-M dynamics | N/A | Insulin dose optimization |
| Colmegna et al., 2020 [37] (J) | T1D | N/A | GLC-I-M dynamics | N/A | Generate data and adjust insulin dose and meal |
| Vaskovsky et al., 2020 [55] (C) | Pre-D | Meal and patient representation | N/A | Personalized nutrition model | Recommend meal and prevent diabetes progression |
| Nowicki, 2021 [67] (C) | T1D | N/A | GLC-I-M dynamics | N/A | Insulin dose optimization |
| Keshary et al., 2022 [60,73] (J) | T2D | N/A | GLC-I-M-exercise dynamics | Meal identification | Insulin dose optimization of AP |
| Silfvergren et al., 2022 [54] (J) | Un-healthy lifestyle | N/A | GLC-I-M dynamics | N/A | Model diet schemes to recommend meals |
| Arefeen and Ghasemzadeh, 2023 [14] (C) | T1D | N/A | N/A | Glucose forecasting, risk identification, and parameter adjustment | Maintain BGL by behavioral changes and education |
| Batagov et al., 2023 [48] (J) | T2D | Metabolic KG and vectorization | Metabolic flux relationships | T2D progression to retinopathy/cataract, and risk stratification | Monitor and prevent T2D progression |
| Cappon et al., 2023 [5] (C) | Pediatric T1D | N/A | GLC-I dynamics [62] | AI-based text suggestions | MDI therapy optimization, recommendation/ telemonitoring |
| Chahal et al., 2023 [57] (C) | Diabetes | N/A | N/A | Classification of diabetic retinopathy, risk stratification | Diagnosis of damaged retina |
| Herrgårdh et al., 2023 [7] (J) | T2D | N/A | I-M-drug dynamics | N/A | Forecast weight changes and insulin resistance, and recommend lifestyle |
| Lee et al., 2023 [9] (J) | T1D, T2D | N/A | Comorbidity progression [97] | Insulin dose and lifestyle recommendation | Monitor patient and adjust lifestyle |
| Paglialonga et al., 2023 [8] (W) | Pre-T2D | N/A | Metabolic and inflammatory dynamics [82,84–86] | Personalized risk assessment and treatment recommendation | Prevent T2D onset |
| Thamotharan et al., 2023 [23] (J) | Elderly T2D | Data aggregation and patient profile generation | Predicting future values | Glucose prediction, insulin dose, food, activity recommendation | Insulin dose optimization and education |
| Wang et al., 2023 [61] (J) | T2D | Patient model with transformer-based vectorization | Define treatment decisions, state transitions, and reward functions for insulin optimization | Optimize insulin, hidden state computation, glycemic states prediction | Insulin dose optimization of AP |
| Zhu et al., 2023 [59] (J) | T1D | N/A | N/A | Glucose time series generation | Increase training data, and enable virtual trials to explore treatments |
| Erdos et al., 2024 [66] (J) | Un- healthy lifestyle, T2D | N/A | GLC-I metabolism [66,77] | N/A | Quantify key parameters and disease prediction |
| Faruqui et al., 2024 [62] (PP) | T2D | N/A | N/A | Glucose and weight predictions, meal recommendations | Maintain glucose and weight by adjusting lifestyle |
| Kalita et al., 2024 [64] (J) | T1D, T2D | N/A | N/A | Glucose forecast | Monitor glucose and insulin dose optimization |
| Koutny, 2024 [70] (J) | T1D | N/A | Input is translated into mechanistic models | N/A | Metabolic language to generate glucose simulations |
| Rad et al., 2024 [47] (J) | T1D, T2D | Patient health KG | Conceptually | Predicting glucose, optimizing insulin dose, lifestyle recommendation | Treatment optimization and maintain euglycemia |
| Sai et al., 2024 [39] (J) | Chronic diseases | N/A | N/A | Disease stage identification and lifestyle recommendation | Treatment optimization |
| Shamanna et al., 2024 [56] (J) | T2D | Patient profile generation | N/A | Predict metabolic health state, glucose peaks, and diet recommendation | Monitor patient progress, medication and meal intake optimization |
| Somers et al., 2024 [65] (PP) | T1D | N/A | GLC-I-liver dynamics model [80] | N/A | Extrapolate unseen glucose behavior |
| Surian et al., 2024 [40] (J) | T2D | Metabolic KG and vectorization | Metabolic flux relationships | Progression from non-CKD to CKD, risk stratification | Monitor risk for CKD onset |
| Young et al., 2024 [24] (J) | T1D | N/A | GLC-exercise dynamics [76] | N/A | Manage exercise schedule and avoid hypoglycemia |
| Zhang et al., 2024 [25] (J) | T2D | N/A | Conceptually | Predict disease progression and contextualize with metabolic KGs | Diagnose progression to T2D |
| de Oliveira et al., 2025 [68] (C) | T2D | N/A | GLC-I dynamics model [77] | N/A | Prevent T2D progression |

Abbreviations

AP: Artificial Pancreas, BGL: Blood glucose levels, CKD: Chronic Kidney Disease, C: Conference Paper, GLC: Glucose, I: Insulin, J: Journal, KG: Knowledge Graph, M: Meal, PP: Preprint, R: Representation, T1D: Type 1 Diabetes, T2D: Type 2 Diabetes, W: Workshop Paper.

**Table S2.** Collected data of included studies.

| Study | Meal data | | | Lab data | | Historical Static data | | | | | Dynamic data | | | |
| --- | --- | --- | --- | --- | --- | --- | --- | --- | --- | --- | --- | --- | --- | --- |
|  | CHO | PRO | Fat | MB | GNT | DG | FH | EHR | IMG | D | PA | GLC | I | VS |
| [69] | ✔ |  |  |  |  |  |  |  |  |  |  | ✔ | ✔ |  |
| [37] | ✔ |  |  |  |  |  |  |  |  |  |  | ✔ | ✔ |  |
| [53] | ✔ | ✔ | ✔ | ✔ | ✔ | ✔ |  | ✔ |  |  | ✔ |  |  |  |
| [55] | ✔ | ✔ | ✔ |  |  |  |  | ✔ |  |  |  |  |  |  |
| [67] | ✔ | ✔ | ✔ |  |  |  |  |  |  |  |  | ✔ | ✔ |  |
| [60] |  |  |  |  |  |  |  |  | ✔ |  | ✔ | ✔ | ✔ | ✔ |
| [54] | ✔ | ✔ |  | ✔ |  | ✔ |  |  |  |  |  |  |  |  |
| [14] | ✔ |  |  |  |  |  |  |  |  |  | ✔ | ✔ | ✔ | ✔ |
| [48] |  |  |  | ✔ |  | ✔ |  |  |  |  |  |  |  | ✔ |
| [5] | ✔ |  |  |  |  |  |  |  |  |  |  | ✔ | ✔ | ✔ |
| [57] |  |  |  |  |  |  |  |  | ✔ |  |  |  |  |  |
| [7] | ✔ |  |  |  |  | ✔ |  |  |  | ✔ |  |  |  |  |
| [9] | ✔ | ✔ | ✔ |  | ✔ |  |  | ✔ |  |  | ✔ | ✔ | ✔ |  |
| [8] | ✔ | ✔ | ✔ |  |  | ✔ |  | ✔ |  | ✔ | ✔ | ✔ |  |  |
| [58] |  |  |  |  |  | ✔ |  | ✔ | ✔ | ✔ |  | ✔ |  | ✔ |
| [23] | ✔ |  |  |  |  |  | ✔ | ✔ |  | ✔ | ✔ | ✔ | ✔ | ✔ |
| [63] | ✔ | ✔ | ✔ |  |  | ✔ |  | ✔ |  |  |  |  |  |  |
| [61] |  |  |  |  |  | ✔ |  | ✔ |  |  |  | ✔ | ✔ |  |
| [59] | ✔ |  |  |  |  |  |  |  |  |  |  | ✔ | ✔ |  |
| [66] |  |  |  |  |  |  |  |  |  |  |  | ✔ | ✔ |  |
| [62] | ✔ | ✔ | ✔ |  |  | ✔ |  |  |  |  | ✔ | ✔ |  |  |
| [64] | ✔ |  |  |  |  |  |  |  |  |  | ✔ | ✔ | ✔ |  |
| [47] | ✔ |  |  |  |  |  |  | ✔ |  |  |  | ✔ | ✔ |  |
| [39] | ✔ |  |  |  |  | ✔ |  |  | ✔ |  | ✔ | ✔ |  | ✔ |
| [56] | ✔ | ✔ | ✔ |  |  | ✔ |  |  | ✔ |  | ✔ |  |  | ✔ |
| [65] | ✔ |  |  |  |  |  |  |  |  |  |  | ✔ | ✔ |  |
| [38] |  | ✔ | ✔ | ✔ |  | ✔ |  |  |  |  |  | ✔ |  |  |
| [24] | ✔ |  |  |  |  |  |  |  |  |  | ✔ | ✔ | ✔ | ✔ |
| [25] |  |  |  | ✔ |  |  |  |  |  |  |  |  |  |  |

Abbreviations

CHO: Carbohydrate, D: Drug, DG: Demographic, EHR: Electronic Health Record, FH: Family History, GLC: Glucose, GNT: Genetic, I: insulin, IMG: Image, MB: Metabolic, PA: Physical Activity, PRO: Protein, VS: Vital Signs.
**Table S3.** Mechanistic processes and their outcome.

| Module | Sub-process(es) | Output(s) |
| --- | --- | --- |
| Insulin | Insulin dynamics on subcutaneous tissue and cellular levels | Appearance of insulin after injection |
|  |  | Absorption dynamics of exogenous insulin infusion to the plasma |
|  |  | Degradation of insulin |
|  | Endogenous insulin production | Effect on glucose uptake |
|  |  | Transition to diabetes |
|  | Insulin resistance on liver and muscle | Effect on glucose uptake |
|  | Insulin kinetics | Appearance of plasma insulin concentration |
| Glucose | Plasma compartment | Oral glucose absorption, rate of glucose appearance in plasma |
|  | Peripheral tissues | Insulin and glucose uptake |
|  | Interstitial fluid and subcuntaenuous tissue | Glucose rate and absorption |
|  |  | Glucose appearance in plasma |
|  |  | Interstitial glucose concentration transported from plasma |
|  | Renal compartment | Renal excretion and utilization of glucose |
|  | Muscle and adipose tissue | Glucose appearance |
|  | Liver compartment | Hepatic glucose production, storage and supply |
|  | Stomach liquid and solid | Glucose rate in liquid and solid states |
|  | Jejunum compartment | Glucose concentration in the intestine |
| Glucagon | Glucagon dynamics | Action of plasma to endogenous glucose production |
|  | Plasma Compartment | Glucagon degradation and plasma glucagon appearance |
| Drug | Tissue level | Response to topiramate |
| Inflammatory system | Effect of calorie intake on adipocytes | Weight gain due to change of volume in adipocytes |
|  | Calorie intake and inflammation | Inflammation activation due to accumulation of adipose tissues |
|  | Interleukin 6 and exercise dynamics | Secretion of Interleukin 6 by adipose and skeletal muscle tissue |
|  |  | Changes in Interleukin 6 |
| Exercise | Oxygen consumption | Watt consumed per minute for walking, running and stepping |
|  | Epipheponrine dynamics | Changes in oxygen consumption during exercise |
|  |  | Changes in epiphrine concentration due to exercise |
|  | Exercise insulin dynamics | Changes in insulin sensitivity |
|  | Activity weight dynamics | Changes in weight |
| Gastrointestinal tract | Weight meal dynamics | Phenomenological energy intake over time |
|  |  | Changes in weight |
|  | Glucose meal or protein meal responses | Glucose appearance after meal |
|  | Gastric emptying | Glucose leaving stomach |
|  |  | Glucose absorption into jejenum |
|  |  | Glucose delivery to ileum |
|  |  | Glucose, alanine and triglycerides appearance rate |
|  | Gut and plasma compartment | Absorption rate of glucose and insulin |
|  | Glucose utilization in fat tissue | Glucose rate of appearance |
| Metabolism | Glucose insulin dynamics and kinetics | Changes in glucose values and glucose mass |
|  |  | Changes in insulin concentrations |
|  |  | Action of plasma insulin on glucose disposal and production |
|  |  | Effect of insulin action and oral glucose rate of appearance on plasma glucose concentration |
|  | Hormonal glucagon insulin dynamics | Epiphrine contribution to the insulin dynamics |
|  |  | Appearance of insulin and glucagon concentrations |
|  | Glucose insulin meal dynamics | Organ specific glucose utilization |
|  |  | Hepatic glycogen storage |
|  |  | Hepatitic protein metabolism utilization |
|  | Glucose insulin glucagon dynamics | Glucose concentration |
|  | Relationship between metabolites and reactions | Evolution of biochemical pathways |
|  | Exercise, glucose, meal dynamics | Insulin dose adjustement |

**Table S4.** Data-driven use cases, their model outcomes, and the approaches used.

| Use case | Model outcome | Data-driven model | Study |
| --- | --- | --- | --- |
| Disease detection | Motif discovery of diagnose patterns | Matrix profile-based analysis | [23] |
|  | Diabetic retinopathy/ cataract | CNN with EfficientNet | [57] |
|  |  | LR | [40] |
|  | CKD | LR | [48] |
| Disease progression | Prediction of chronic wound healing time | Image processing and AI models | [58] |
|  | Biomarker evolution relevant to diabetes | MIMO Gaussian process model with an auto-regressive structure | [8] |
|  | Disease progression | Regression with LassoCV model | [25] |
|  |  | Classification with LR | [25] |
|  | Disease state detection | Support Vector Machine | [39] |
| Glucose regulation | Glucose forecasting | LSTM | [23] |
|  |  | Adaptable semi parametrized (linear) regression | [23] |
|  |  | CatBoostRegressor + RF + LSTM | [56] |
|  |  | RNN | [47] |
|  |  | Hybrid LSTM + GRU | [64] |
|  |  | Stacked CNN LSTM model | [14] |
|  |  | MLP/ANN | [61,62] |
|  | Synthetic glucose time series generation | GANs and RNNs | [59] |
|  | Personalized glucose regulation | RL: Soft Actor Critic Algorithm | [47] |
| Lifestyle detection | Activity classification | Threshold comparison-based classifier | [39] |
|  | Weight forecasts | MLP/ANN | [62] |
| Insulin infusion | Personalized insulin infusion | LB MPC | [23] |
|  | Dynamic insulin infusion | RL | [61] |
| Treatment recommendation | Personal lifestyle recommendation | Support Vector Description Classifier | [8] |
|  | Drug recommendation | RF and LightGBM | [39] |
|  | Wound treatment plan generation | Image processing and AI models | [58] |
|  | Meal recommendation | Image classification | [39] |
|  |  | Logic Rules and Reasoning embedded within the KGs | [47] |
|  |  | ANN | [62] |
|  |  | Logical inference machine | [74] |
| XAI | Explain simulation outcome | Counterfactual Explanations | [8] |
|  | Explanations of hyper- and hypoglycemia | GradientBoost with LIME | [23] |
|  | Explore impact of features | Gradient weighted class activation mapping | [14] |

Abbreviations

AI: Artificial Intelligence, ANN: Artificial Neural Network, CNN: Convolutional Neural Network, GAN: Generative Adversarial Network, GRU: Gated Recurrent Unit, KG: Knowledge Graph, LassoCV: Least Absolute Shrinkage and Selection Operator with Cross-Validation, LB-MPC: Learning-Based Model Predictive Control, LightGBM: Light Gradient Boosting Machine, LIME: Local Interpretable Model-Agnostic Explanations, LR: Logistic Regression, LSTM: Long Short-Term Memory, MIMO: Multiple Input Multiple Output, MLP: Multilayer Perceptron, RF: Random Forest, RNN: Recurrent Neural Network, XAI: Explainable AI.

**References**

69. Goodwin GC, Seron MM, Medioli AM, Smith T, King BR, Smart CE. A systematic stochastic design strategy achieving an optimal tradeoff between peak BGL and probability of hypoglycaemic events for individuals having type 1 diabetes mellitus. Biomed Signal Process Control. Mar 2020;57:101813. [doi: 10.1016/j.bspc.2019.101813]

37. Colmegna P, Wang K, Garcia-Tirado J, Breton MD. Mapping data to virtual patients in type 1 diabetes. Control Eng Pract. Oct 2020;103:104605. [doi: 10.1016/j.conengprac.2020.104605]

55. Vaskovsky AM, Chvanova MS, Rebezov MB. Creation of digital twins of neural network technology of personalization of food products for diabetics. Presented at: 2020 4th Scientific School on Dynamics of Complex Networks and their Application in Intellectual Robotics (DCNAIR); Sep 7-9, 2020:251-253; Innopolis, Russia. [doi: 10.1109/ DCNAIR50402.2020.9216776]

67. Nowicki T. Virtual therapy using type 1 diabetes direct simulator. J Phys: Conf Ser. Jan 1, 2021;1736(1):012031. [doi: 10.1088/1742-6596/1736/1/012031]

60. Keshary S, T P, Bekiroglu K, Seshadhri S, Srinivasan S. Multimedia data-based artificial pancreas for type 2 diabetes. IEEE MultiMedia. 2022;29(1):18-27. [doi: 10.1109/MMUL.2022.3154534]

73. Lenatti M, Carlevaro A, Guergachi A, Keshavjee K, Mongelli M, Paglialonga A. A novel method to derive personalized minimum viable recommendations for type 2 diabetes prevention based on counterfactual explanations. PLoS ONE. 2022;17(11):e0272825. [doi: 10.1371/journal.pone.0272825] [Medline: 36395096]

54. Silfvergren O, Simonsson C, Ekstedt M, Lundberg P, Gennemark P, Cedersund G. Digital twin predicting diet response before and after long-term fasting. PLoS Comput Biol. Sep 2022;18(9):e1010469. [doi: 10.1371/journal.pcbi.1010469] [Medline: 36094958]

14. Arefeen A, Ghasemzadeh H. GlySim: modeling and simulating glycemic response for behavioral lifestyle interventions. Presented at: 2023 IEEE EMBS International Conference on Biomedical and Health Informatics (BHI); Oct 15-18, 2023:1-5; Pittsburgh, PA. [doi: 10.1109/BHI58575.2023.10313443]

48. Batagov A, Dalan R, Wu A, Lai W, Tan CS, Eisenhaber F. Generalized metabolic flux analysis framework provides mechanism-based predictions of ophthalmic complications in type 2 diabetes patients. Health Inf Sci Syst. Dec 2023;11(1):18. [doi: 10.1007/s13755-023-00218-x] [Medline: 37008895]

5. Cappon G, Pellizzari E, Cossu L, et al. System architecture of TWIN: a new digital TWIN-based clinical decision support system for type 1 diabetes management in children. Presented at: 2023 IEEE 19th International Conference on Body Sensor Networks (BSN); Oct 9-11, 2023:1-4; Boston, MA. [doi: 10.1109/BSN58485.2023.10331272]

57. Chahal Y, Tokas R, Sharma K. Smart solution using digital twin and IoT for diabetic retinopathy. Presented at: 2023 14th International Conference on Computing Communication and Networking Technologies (ICCCNT); Jul 6-8, 2023:1-6; Delhi, India. [doi: 10.1109/ICCCNT56998.2023.10307620]

7. Herrgårdh T, Simonsson C, Ekstedt M, et al. A multi-scale digital twin for adiposity-driven insulin resistance in humans: diet and drug effects. Diabetol Metab Syndr. Dec 4, 2023;15(1):250. [doi: 10.1186/s13098-023-01223-6] [Medline: 38044443]

9. Lee J, Yu J, Yoon KH. Opening the precision diabetes care through digital healthcare. Diabetes Metab J. May 2023;47(3):307-314. [doi: 10.4093/dmj.2022.0386] [Medline: 36977545]

8. Paglialonga A, Lenatti M, Simeone D, et al. Towards a digital twin for personalized diabetes prevention: the PRAESIIDIUM project. Presented at: BUILD-IT 2023 Workshop (Building a Digital Twin: Requirements, Methods, and Applications); Oct 19-20, 2023; Rome, Italy. URL: https://iris.cnr.it/retrieve/3d3f267e-bf3d-4ed2-8276- beea33db8351/prod_488661-doc_203317.pdf [Accessed 2026-09-16]

23. Thamotharan P, Srinivasan S, Kesavadev J, et al. Human digital twin for personalized elderly type 2 diabetes management. J Clin Med. Mar 7, 2023;12(6):2094. [doi: 10.3390/jcm12062094] [Medline: 36983097]

61. Wang G, Liu X, Ying Z, et al. Optimized glycemic control of type 2 diabetes with reinforcement learning: a proof-ofconcept trial. Nat Med. Oct 2023;29(10):2633-2642. [doi: 10.1038/s41591-023-02552-9] [Medline: 37710000]

59. Zhu T, Li K, Herrero P, Georgiou P. GluGAN: generating personalized glucose time series using generative adversarial networks. IEEE J Biomed Health Inf. Oct 2023;27(10):5122-5133. [doi: 10.1109/JBHI.2023.3271615] [Medline: 37134028]

66. Erdős B, O’Donovan SD, Adriaens ME, et al. Leveraging continuous glucose monitoring for personalized modeling of insulin-regulated glucose metabolism. Sci Rep. Apr 5, 2024;14(1):8037. [doi: 10.1038/s41598-024-58703-6] [Medline: 38580749]

62. Faruqui SHA, Alaeddini A, Du Y, Li S, Sharma K, Wang J. Nurse-in-the-loop artificial intelligence for precision management of type 2 diabetes in a clinical trial utilizing transfer-learned predictive digital twin (preprint). JMIR Preprints. Preprint posted online on Mar 14, 2024. [doi: 10.2196/preprints.58412]

64. Kalita D, Sharma H, Panda JK, Mirza KB. Platform for precise, personalised glucose forecasting through continuous glucose and physical activity monitoring and deep learning. Med Eng Phys. Oct 2024;132:104241. [doi: 10.1016/j. medengphy.2024.104241] [Medline: 39428134]

70. Koutny T. Sirael: virtual metabolic machine. Biomed Mater Devices. Mar 2025;3(1):576-585. [doi: 10.1007/s44174- 024-00199-9]

47. Rad FS, Hendawi R, Yang X, Li J. Personalized diabetes management with digital twins: a patient-centric knowledge graph approach. J Pers Med. Mar 28, 2024;14(4):359. [doi: 10.3390/jpm14040359] [Medline: 38672986]

39. Sai S, Gaur A, Hassija V, Chamola V. Artificial intelligence empowered digital twin and NFT-based patient monitoring and assisting framework for chronic disease patients. IEEE Internet Things M. 2024;7(2):101-106. [doi: 10.1109/IOTM. 001.2300138]

56. Shamanna P, Joshi S, Thajudeen M, et al. Personalized nutrition in type 2 diabetes remission: application of digital twin technology for predictive glycemic control. Front Endocrinol (Lausanne). 2024;15:1485464. [doi: 10.3389/fendo.2024. 1485464] [Medline: 39634180]

65. Somers R, Walkinshaw N, Hierons RM, Elliott J, Iqbal A, Walkinshaw E. Configuration testing of an artificial pancreas system using a digital twin: an evaluative case study. J Software Test, Verif Reliab. Feb 2025;35(2):e70000. [doi: 10. 1002/stvr.70000]

40. Surian NU, Batagov A, Wu A, et al. A digital twin model incorporating generalized metabolic fluxes to identify and predict chronic kidney disease in type 2 diabetes mellitus. npj Digit Med. May 24, 2024;7(1):140. [doi: 10.1038/s41746- 024-01108-6] [Medline: 38789510]

24. Young G, Dodier R, Youssef JE, et al. Design and In Silico Evaluation of an Exercise Decision Support System Using Digital Twin Models. J Diabetes Sci Technol. Mar 2024;18(2):324-334. [doi: 10.1177/19322968231223217] [Medline: 38390855]

25. Zhang Y, Qin G, Aguilar B, et al. A framework towards digital twins for type 2 diabetes. Front Digit Health. 2024;6:1336050. [doi: 10.3389/fdgth.2024.1336050] [Medline: 38343907]

68. de Oliveira CD, James L, Khanshan A, Van Gorp P. Toward enhancing diabetes self-management with personalization through human digital twins for behavior change in: lecture notes in networks and systems. Presented at: Proceedings of Ninth International Congress on Information and Communication Technology: ICICT 2024; Feb 19-22, 2024:623-634; London. [doi: 10.1007/978-981-97-5035-1_49]

53. Gkouskou K, Vlastos I, Karkalousos P, Chaniotis D, Sanoudou D, Eliopoulos AG. The “virtual digital twins” concept in precision nutrition. Adv Nutr. Nov 16, 2020;11(6):1405-1413. [doi: 10.1093/advances/nmaa089] [Medline: 32770212]

Silfvergren O, Simonsson C, Ekstedt M, Lundberg P, Gennemark P, Cedersund G. Digital twin predicting diet response before and after long-term fasting. PLoS Comput Biol. Sep 2022;18(9):e1010469. [doi: 10.1371/journal.pcbi.1010469] [Medline: 36094958]

58. Sarp S, Kuzlu M, Zhao Y, et al. Digital twin in chronic wound management. In: Karaarslan E, Aydin Ö, Cali Ü, Challenger M, editors. Digital Twin Driven Intelligent Systems and Emerging Metaverse. Springer Nature Singapore; 2023:233-248. [doi: 10.1007/978-981-99-0252-1_11]

63. Vahdati M, Saghiri AM, HamlAbadi KG. Digital twins for nutrition. In: Digital Twin for Healthcare. Elsevier; 2023:305-323. [doi: 10.1016/B978-0-32-399163-6.00020-2]

38. He Q, Li L, Li D, et al. From digital human modeling to human digital twin: framework and perspectives in human factors. Chin J Mech Eng. 2024;37(1):9. [doi: 10.1186/s10033-024-00998-7]

74. Vaskovsky AM, Chvanova MS. Designing the neural network for personalization of food products for persons with genetic president of diabetic sugar. Presented at: 2019 3rd School on Dynamics of Complex Networks and their Application in Intellectual Robotics (DCNAIR); Sep 9-11, 2019:175-177; Innopolis, Russia. [doi: 10.1109/DCNAIR. 2019.8875559]
